# Phenotypic and genotypic characterization of *Marinobacterium weihaiense* sp. nov. and *Marinobacterium marinum* sp. nov., isolated from marine sediment, and genomic properties of the genus *Marinobacterium*

**DOI:** 10.1099/mgen.0.001182

**Published:** 2024-01-24

**Authors:** Xin-Jiang Liu, Ke-Lei Zhu, Yu-Qi Ye, Ze-Tian Han, Xin-Yun Tan, Zong-Jun Du, Meng-Qi Ye

**Affiliations:** ^1^​ Shenzhen Research Institute of Shandong University, Shenzhen, Guangdong, 518057, PR China; ^2^​ Marine College, Shandong University, Weihai, Shandong, 264209, PR China; ^3^​ Weihai Research Institute of Industrial Technology of Shandong University, Weihai, 264209, PR China

**Keywords:** comparative genomic analysis, *Marinobacterium*, polyhydroxyalkanoates, polyphasic taxonomy

## Abstract

In this study, two novel bacterial strains were isolated from coastal sediment of Weihai, China. The two strains were Gram-stain-negative and facultatively aerobic, designated 3-1745^T^ and A346^T^. Based on phenotypic, genetic and phylogenetic properties, strains 3-1745^T^ and A346^T^ represent two novel species of the genus *Marinobacterium*. The results of genome analysis revealed many central carbohydrate metabolism pathways such as gluconeogenesis, pyruvate oxidation, tricyclic acid cycle, pentose phosphate pathway and PRPP biosynthesis in the genus *Marinobacterium*. The ability of strains 3-1745^T^ and A346^T^ to utilize volatile fatty acids was experimentally confirmed. Polyhydroxyalkanoate synthases (PhaA, PhaB and PhaC) for the synthesis of polyhydroxyalkanoates were prevalent in the genus *Marinobacterium*. Multiple BGCs (biosynthetic gene clusters) including betalactone, ectoine, ranthipeptide, redox-cofactor, RiPPs (ribosomally synthesized post-translationally modified peptides) and T3PKS (polyketide synthases) in the genome of the genus *Marinobacterium* were found. Additional genome analyses suggested that the genus *Marinobacterium* contained diverse potential mechanisms of salt tolerance and mainly utilized oligosaccharides. This is the first report on broad genomic analyses of the genus *Marinobacterium* with the description of two novel species and potential ecological and biotechnological implications.

## Abbreviations

AAI, average amino acid identity; ANI, average nucleotide identity; BGC, biosynthetic gene cluster; CAZyme, carbohydrate-active enzyme; DDH, DNA–DNA hybridization; hserlactone, homoserine lactone; LAP, linear azol(in)e-containing peptides; ML, maximum-likelihood; PHA, polyhydroxyalkanoate; PRPP, phosphoribosyl diphosphate; RiPPs, ribosomally synthesized post-translationally modified peptides; SOX, sulphur-oxidizing; T3PKS, polyketide synthases.

## Impact Statement

The genus *Marinobacterium* belongs to the family *Oceanospirillaceae* of the class *Gammaproteobacteria* in the phylum *Pseudomonadota*. Most members of the genus *Marinobacterium* have been isolated from different marine environments, such as seawater, sediment, coral, tidal flat and mangrove roots. In this work, we studied two strains isolated from marine sediment. Based on phenotypic, genetic and phylogenetic properties, strains A346^T^ and 3-1745^T^ represent two novel species of the genus *Marinobacterium*. Comparative genome analysis was conducted to study their properties and functions. Strains A346^T^ and 3-1745^T^ have potential to produce polyhydroxyalkanoates and harbour multiple secondary metabolites (biosynthetic gene clusters). They can oxidize thiosulphate to produce sulphate and may play roles in sulphur cycling. In this study, the isolation and identification of strains A346^T^ and 3-1745^T^ further enriched the marine microbial resources and gene library. The genus *Marinobacterium* has complete nitrogen fixation, dissimilatory nitrate reduction and denitrification pathways, which might foster future projects to develop and utilize the strains industrially. Our understanding of the genus *Marinobacterium* with regard to metabolic pathways and multiple potential mechanisms of salt tolerance was also deepened.

## Data Summary

This study generated sequencing data for strains A346^T^ and 3-1745^T^ and all sequence data have been uploaded to the National Center for Biotechnology Information. The GenBank accession number for the 16S rRNA gene sequence and draft genome of strain A346^T^ is MZ434947 and JAHQZT000000000. The GenBank accession number for the 16S rRNA gene sequence and draft genome of strain 3-1745^T^ is MW391814 and JACEMT000000000. Detailed information can be found in Table S1 (available in the online version of this article).

## Introduction

The genus *Marinobacterium* was first described in 1997, and was isolated from marine pulp mill effluent enrichment cultures [1]. The genus *Marinobacterium* widely exists in a variety of habitats; 18 species of the genus have been isolated from different environments, such as seawater [2], sediment [3], coral [4], tidal flat [5], mangrove roots [6] and high-salinity soil [7]. In addition, the genus *Marinobacterium* has also been detected in other samples including marine sediments taken from the Yellow Sea of South Korea [8], oil seep marine sediments from the southern Gulf of Mexico [9], rhizosphere soil from India [10], and different oil fields from Brazil, Malaysia and China [11–13]. The genome analysis results in this study suggest that the genus *Marinobacterium* contains diverse potential mechanisms of salt tolerance, and thus halotolerance is a general characteristic of members of this genus.

Some members of the genus *Marinobacterium* are capable of nitrogen-fixation [6], benzene-degradation [14], producing antimicrobial and cytotoxic ortho-dialkylbenzene-class metabolites [15], and the decomposition of marine aromatic compounds [16]. It has been reported that members of this genus can produce polyhydroxyalkanoates (PHAs) from various carbohydrates and volatile fatty acids [17]. PHAs have a variety of medical applications: biocontrol agents, drug carriers, biodegradable implants, anti-osteoporosis agents, medical devices, antibacterials, tissue engineering, memory enhancers and anticancer agents [18].

In order to further enrich marine microbial resources and understand their survival mechanism and application potentialities, our laboratory has carried out research on the mining of microbial resources. In this study, we collected coastal intertidal sediments from Xiaoshi Island, Weihai, China, which is located in a National Special Marine Reserve with a good degree of protection and little human exploitation. Measured salinity and pH values of the coastal sediment samples were 25‰ and 7.5, respectively. Two new strains of the genus *Marinobacterium* were isolated from marine sediments, the taxonomic positions of strains A346^T^ and 3-1745^T^ were determined by exploring phenotypic, genetic and phylogenetic properties, and comparative genome analysis was conducted to study their properties and potential functions. The two newly isolated strains had a short growth cycle, obvious colony formation within 1 day of inoculation, and were able to survive in a highly saline and alkaline environment, and through genomic analysis of the genus *Marinobacterium*, members of this group have the potential to produce PHAs. Reducing sulphides can be utilized by a variety of bacteria, the most commonly known being sulphur-oxidizing bacteria of the genera *Thiobacillus* [19], *Thiomicrospira* [20] and *Thiothrix* [21]. To date, thiosulphate oxidation in the genus *Marinobacterium* has not been reported. The current study showed that the genus *Marinobacterium* has the potential to oxidize thiosulphate and may participate in nitrogen fixation, dissimilatory nitrate reduction and denitrification, indicating that members of the genus may play an important role in geochemical cycles.

## Methods

### Bacterial isolation and identification

Strains A346^T^ and 3-1745^T^ were isolated from coastal sediment taken from Weihai, China (37° 31′ 3″ N 122° 1′ 6″ E). Sample pore water was extracted using a Rhizonsphere solution sampler (https://www.rhizosphere.com/rhizons) to determine sample salinity. Temperature and pH were measured by using a digital display rapid thermometer (TM902C; Qiankuang) and portable pH meter (PHB-5; Qiwei). Measured salinity, temperature and pH of the coastal sediment samples were 25 ‰, 20 °C and 7.5, respectively. The sediment sample was continuously diluted and resuspended in sterile seawater. Marine agar 2216 (MA; BD) spread with 10-fold diluted samples was incubated at 30 °C for 7 days. Strains A346^T^ and 3-1745^T^, which formed yellowish-white, circular, shiny colonies, were picked and purified. They were stored at −80 °C in sterile 1 % (w/v) saline supplemented with 15 % (v/v) glycerol. The genomes of strains A346^T^ and 3-1745^T^ were extracted by using a DNA extraction kit (Takara). Details of the PCR conditions for amplification of the 16S rRNA gene were as given in Weisburg *et al*. [22, 23].

Almost-complete 16S rRNA gene sequences were obtained by cloning with pMD18-T vector (Takara) and sequencing primers M13F and M13R as previously described [24]. Sequencing was conducted by BGI (Qingdao, PR China). The nearly complete 16S rRNA gene sequences of strains A346^T^ (1541 bp, with GenBank accession number MZ434947.1) and 3-1745^T^ (1503 bp, with GenBank accession number MW391814.1) were obtained. The level of 16S rRNA gene sequence similarity between strains A346^T^ and 3-1745^T^ was calculated using the EzBioCloud (https://www.ezbiocloud.net/) [25] and NCBI databases (https://www.ncbi.nlm.nih.gov/). The nucleotide collections (nr) from the Standard database (NCBI databases) was used in BLASTn similarity searches. To determine their exact taxonomic status, we performed further phylogenetic and taxonomic studies. Phylogenetic trees were established by the maximum-likelihood (ML) [26] algorithm in the software package mega v11.0 [27], and all bootstrap values were based on 1000 replications. The ML tree was reconstructed using the best-fit substitution model GTR+G+I. The phylogenetic relationship based on a group of 120 conserved genes was analysed via GTDB-Tk [28], and the phylogenetic trees were reconstructed using IQ-TREE [29] with the LG+F+I+G4 model and 1000 bootstrap replicates, and against the GTDB database [30] to confirm the species novelty.

*Marinobacterium stanieri* DSM 7027^T^ (obtained from Deutsche Sammlung von Mikroorganismen und Zellkulturen, DSMZ), *Marinobacterium georgiense* JCM 21667^T^ (obtained from Japan Collection of Microorganisms, JCM) and *Marinobacterium maritimum* JCM 15134^T^ (obtained from JCM) were used as reference strains. *M. georgiense* is the type species of the genus *Marinobacterium. M. stanieri* DSM 7027^T^ and *M. maritimum* JCM 15134^T^ have a close phylogenetic relationship with strains 3-1745^T^ and A346^T^, respectively.

### Genomic analysis

The genomes of strains A346^T^ and 3-1745^T^ were sequenced on the Hiseq X Ten platform (Illumina) at Beijing Novogene Bioinformatics Technology, using the pair-end 350 bp sequencing protocol. The raw data were filtered by fastp software [31] and all cleaned data were assembled by SOAPdenovo software v2.04 [32]. The quality of the assembled genomes was evaluated by using CheckM (v1.1.6) [33]. The genomes of other related strains were downloaded from the NCBI Prokaryotic reference genomes database. The draft genome content was annotated using the NCBI Prokaryotic Genome Annotation Pipeline (PGAP). To further confirm the taxonomic status of strains A346^T^ and 3-1745^T^, the average nucleotide identity (ANI) was calculated using online tools of the EzBioCloud database (https://www.ezbiocloud.net/tools/ani) [34], DNA–DNA hybridization (DDH) values wee calculated by using the Genome-to-Genome Distance Calculator v2.1 [35], and the average amino acid identity (AAI) was calculated by CompareM (https://github.com/dparks1134/CompareM). To investigate the metabolic pathways and potential functions of bacteria, genomes were annotated using the KEGG database (v3.0) [36]. Bacterial resistance genes were annotated through RAST databases (v2.0), Proksee (https://proksee.ca/) [37] and the Comprehensive Antibiotic Resistance Database (CARD) (v3.2.4) [38, 39]. Clusters of orthologous groups (COGs) were annotated in eggNOG-mapper (v2.1.12) [40]. Prediction of secondary metabolites from draft genomes were conducted by using the antiSMASH 6.0 database [41]. Carbohydrate-active enzymes were annotated by the dbCAN2 meta server [42]. Pan-genomic analysis of bacteria was performed by BPGA v1.3 [43], determining core, accessory and unique gene numbers. The percentage of COG and KEGG categories of genes were calculated based on protein blast against reference COG and KEGG databases.

### Phenotypic, physiological and biochemical characteristics

To examine phenotypic and physiological characteristics, the strains were cultured at 35 °C on MA plates. Both strains A346^T^ and 3-1745^T^ were able to grow at 35℃ and grew rapidly at this temperature. After incubation for 3 days, Gram-staining was examined using the method described previously described [44]. Determination of catalase activity was conducted by observing bubble production in 3 % (v/v) H_2_O_2_. Oxidase was determined by an oxidase reagent kit (bioMérieux) according to the manufacturer’s instructions. Cell morphology and size were assessed by using a light microscope (E600; Nikon), a scanning electron microscope (model Nova NanoSEM450; FEI) and a transmission electron microscope (JEM-1200; JEOL). Optimum temperatures for growth were tested at various temperatures (0, 4, 10, 15, 20, 25, 28, 30, 33, 35, 37, 40, 43, 44, 45 and 46 °C) on MA plates. Growth at different NaCl concentrations (0, 0.5, 1.0, 1.5, 2.0, 2.5, 3.0, 4.0, 5.0, 7.0, 8.0, 10.0, 12.0, 14.0, 16.0, 18.0, 19.0 and 20.0 %, w/v) was observed using a medium (0.5 % peptone, 0.1 % yeast extract and 2 % agar), prepared with artificial seawater (MgCl_2_ 0.23 %, MgSO_4_ 0.32 %, CaCl_2_ 0.12 %, KCl 0.07 % and NaHCO_3_ 0.02 %; all w/v) [45]. Growth at various pH (from pH 5.5 to 9.5, in increments of 0.5 pH units) was determined on Marine Broth 2216 (MB; BD). pH values were adjusted with buffers containing MES (pH 5.5–6.0), PIPES (pH 6.5–7.0), HEPES (pH 7.5–8.0), Tricine (pH 8.5) and CAPSO (pH 9.0–14.0) at 35 °C. Cell motility was determined on modified MA medium with 0.3 % agar. The abilities to hydrolyse DNA, starch, agar, casein, alginate, CM-cellulose and Tweens (20, 40, 60 and 80) were tested according to classical methods in bacterial taxonomy [44]. For testing anaerobic growth, strains were grown in anaerobic (10 % H_2_, 10 % CO_2_ and 80 % N_2_) and aerobic (5 % O_2_, 10 % CO_2_ and 85 % N_2_) environments on MA medium with or without 0.1 % (w/v) KNO_3_ for 2 weeks at 35 °C in an anaerobic jar. Antibiotic susceptibility tests of strains A346^T^ and 3-1745^T^ were performed on MA plates at 35 °C for up to 5 days using the disc-diffusion method [46]. Physiological and biochemical characteristics were described by using the results from API 20E kit (bioMérieux), API 50CHB kit (bioMérieux), API ZYM kits (bioMérieux) and Biolog GEN III MicroPlates according to the manufacturers’ instructions. Expression of the sulphur-oxidizing (SOX) system was tested by using a medium (0.5 % peptone, 0.1 % yeast extract and 0.03 % sodium pyruvate), prepared with artificial seawater (MgCl_2_ 0.23 %, MgSO_4_ 0.32 %, CaCl_2_ 0.12 %, KCl 0.07 % and NaS_2_O_4_ 0.5 %, all w/v). S_2_O_3_^2−^ and SO_4_^2−^ were detected by FeCl_3_ and BaCl_2_ solutions at 0 and 7 days [47].

### Chemotaxonomic analyses

To determine chemotaxonomic characteristics, cells of strains A346^T^ and 3-1745^T^, *M. georgiense* JCM 21667^T^, *M. stanieri* DSM 7027^T^ and *M. maritimum* JCM 15134^T^ were cultured in MB for 2 days at 30 °C, and then cells were collected and made into lyophilized powder. Isoprenoid quinones were determined using HPLC [48]. The extraction of polar lipids was extracted using a solution consisting of chloroform, methanol, and water (2.5:5:2, v/v/v) and the spilt off in two different directions on TLC plates according to a previously published method [49]. Fatty acids were obtained and detected using HPLC after adding 50 mg freeze-dried powder and a series of reagents into a 10 ml glass tube, followed by saponification, methylation, extraction and alkali washing [50].

## Results

### Bacterial isolation and identification

**Fig. 1.**
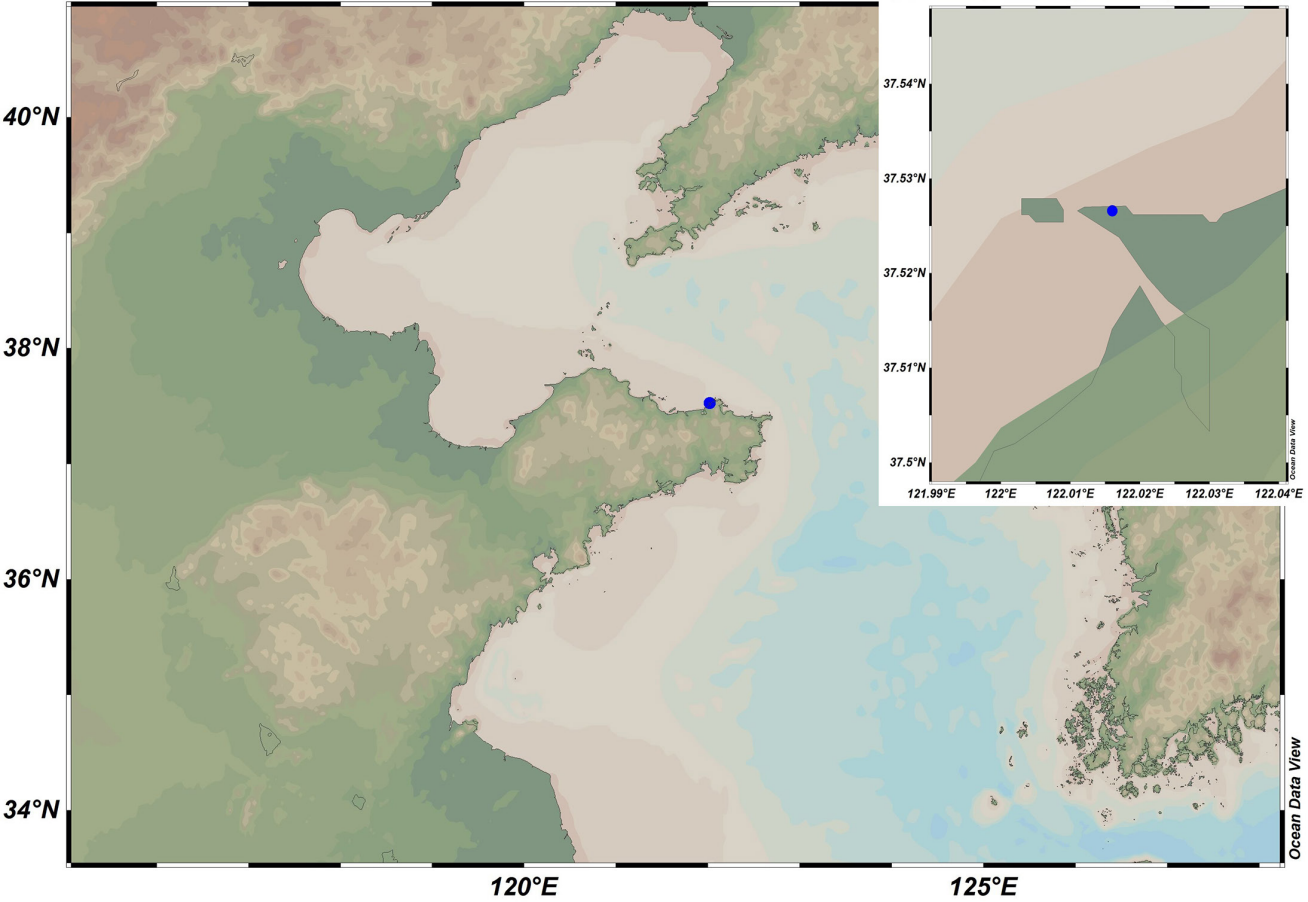
Map showing the sample collection site. The blue dot represents the sampling site, and the upper right corner shows an enlarged image of the sample collection site.

Strains A346^T^ and 3-1745^T^, showing yellowish-white, circular and shiny colonies, were isolated from coastal sediment taken from Weihai, China (Fig. 1). The purified culture was stored at −80 °C in sterile 1 % (w/v) saline supplemented with 15 % (v/v) glycerol.

**Fig. 2.**
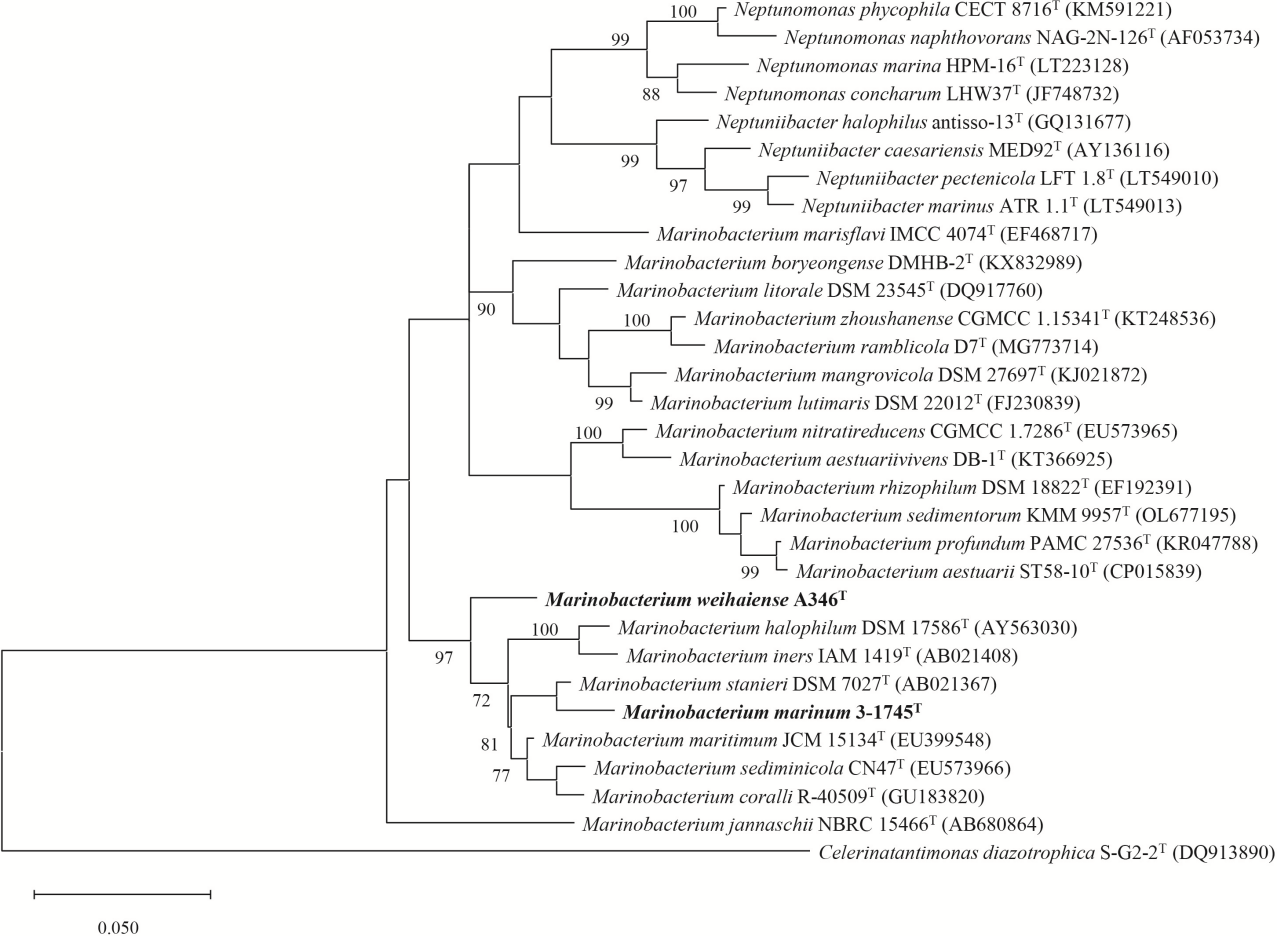
Maximum-likelihood phylogenetic tree based on 16S rRNA gene sequences showing the position of strains A346^T^ and 3-1745^T^. Bootstrap values >70 % are shown at branch nodes. *Celerinatantimonas diazotrophica* S-G2-2^T^ (DQ913890.1) was used as an outgroup. The 16S rRNA gene sequences of strains A346^T^ and 3-1745^T^ were extracted from the genome. Bar, 0.050 substitutions per nucleotide position.

**Fig. 3.**
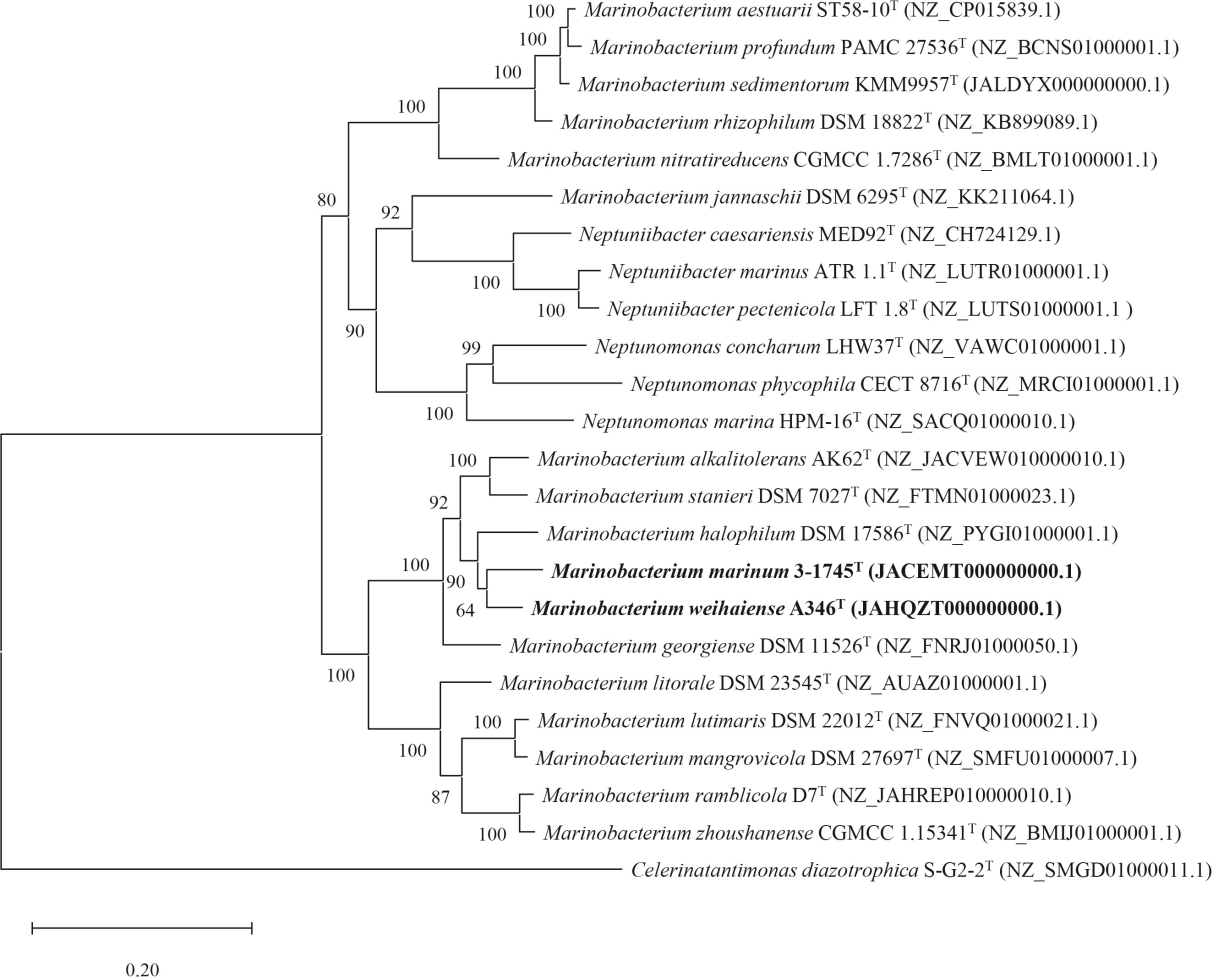
IQ-TREE based on a group of 120 conserved genes showing the relationships between strains A346^T^, 3-1745^T^ and related taxa. Bootstrap values >70 % are shown at branch nodes. *Celerinatantimonas diazotrophica* S-G2-2^T^ (NZ_SMGD01000011.1) was used as an outgroup. Bar, 0.20 substitutions per nucleotide position.

**Fig. 4.**
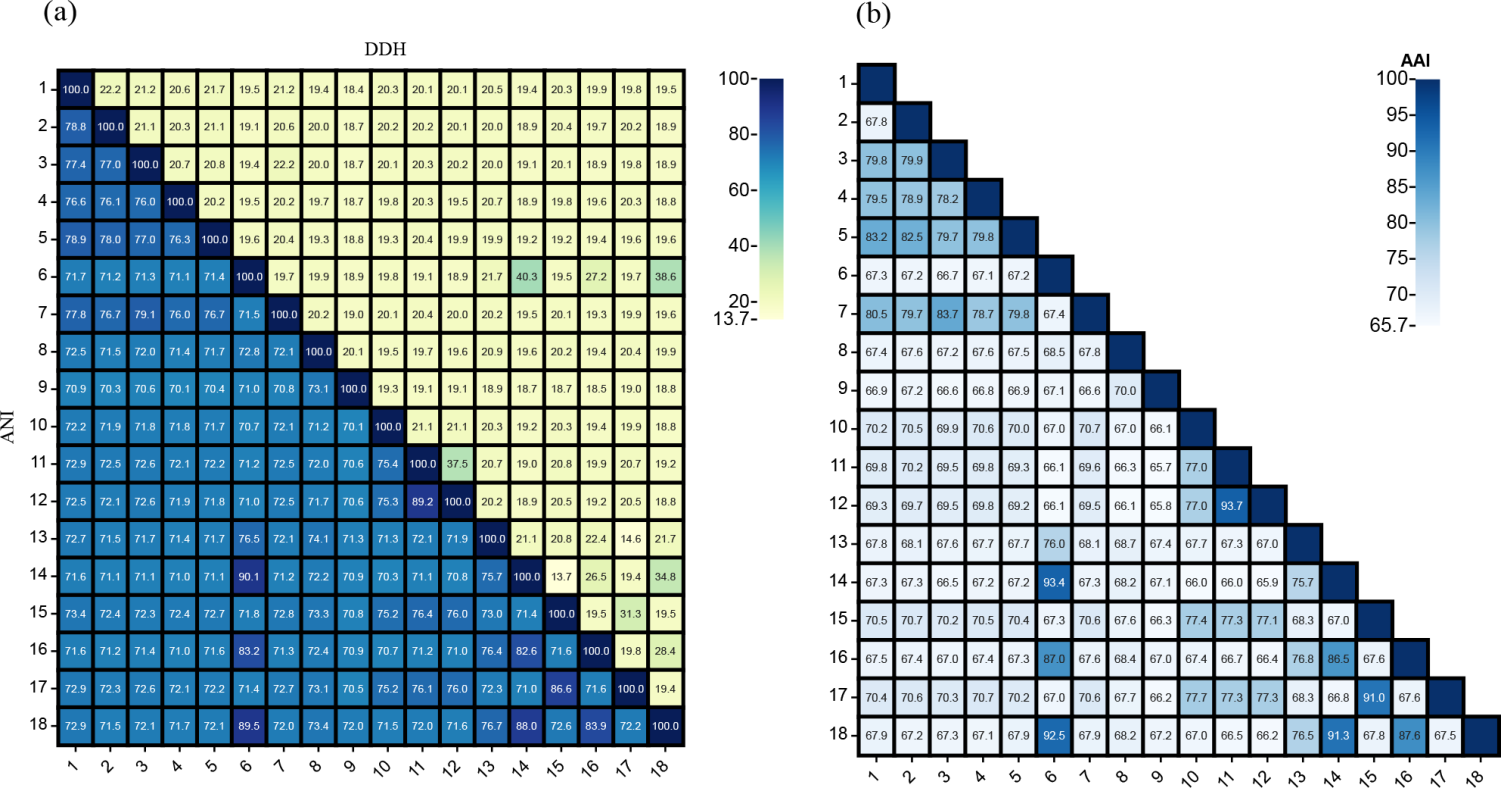
Comparisons of the average nucleotide identity (ANI), DNA–DNA hybridization (DDH) and average amino acid identity (AAI) values between strains A346^T^, 3-1745^T^ and related *Marinobacterium* type strains. (**a**) ANI and DDH values; (**b**) AAI values. Strains: 1, *M. weihaiense* A346^T^; 2, *M. marinum* 3-1745^T^; 3, *M. stanieri* DSM 7027^T^; 4, *M. georgiense* JCM 21667^T^; 5, *M. halophilum* DSM 17586^T^; 6, *M. aestuarii* ST58-10^T^; 7, *M. alkalitolerans* AK62^T^; 8, *M. arenosum* CAU 1594^T^; 9, *M. jannaschii* DSM 6295^T^; 10, *M. litorale* DSM 23545^T^; 11, *M. lutimaris* DSM 22012^T^; 12, *M. mangrovicola* DSM 27697^T^; 13, *M. nitratireducens* CGMCC 1.7286^T^; 14, *M. profundum* PAMC 27536^T^; 15, *M. ramblicola* D7^T^; 16, *M. rhizophilum* DSM 18822^T^; 17, *M. zhoushanense* CGMCC 1.15341^T^; 18, *M. sedimentorum* KMM 9957^T^.

Nearly full-length 16S rRNA gene sequences of strains A346^T^ (1541 bp) and 3-1745^T^ (1503 bp) were obtained. Based on comparison against the EzBioCloud database, both strains A346^T^ and 3-1745^T^ had the highest 16S rRNA gene sequence similarity to *M. stanieri* DSM 7027^T^ (97.0 and 98.4 %, respectively). The 16S rRNA gene sequence similarity between strain A346^T^ (3-1745^T^) and other species of the genus *Marinobacterium* were 92.3–97.0 % (92.4–98.4 %). The 16S rRNA gene sequence similarity between the sediment-derived strains and other members of the genus *Marinobacterium* are detailed in Table S1. According to the results of 16S rRNA gene sequence analysis, the threshold between different bacterial species is 98.7 % [51]. The phylogenetic tree reconstructed with the ML algorithm indicated that strains A346^T^ and 3-1745^T^ were affiliated to the genus *Marinobacterium* (Fig. 2). The 16S rRNA gene sequence similarity between strains A346^T^ and 3-1745^T^ was 96.5 % and they were thus considered to represent two different novel species. Strain A346^T^ was more closely related to *M. maritimum* JCM 15134^T^, and strain 3-1745^T^ was more closely related to *M. stanieri* DSM 7027^T^ in the 16S rRNA gene phylogenetic tree. The genomic phylogenetic tree also showed that both strains A346^T^ and 3-1745^T^ were affiliated to the genus *Marinobacterium* (Fig. 3). DDH, ANI and AAI values are listed in Fig. 4. It was clear that the DDH values were all <41 % and the ANI values were all <91%, which were lower than the species delineation thresholds (70 % for DDH and 95–96 % for ANI) [52, 53], indicating that strains A346^T^ and 3-1745^T^ represent two novel species of the genus *Marinobacterium*. The AAI values between all genomes were >65.7 %, which were higher than the genus delineation thresholds (65 % for AAI) [54], indicating that they were affiliated to the same genus. However, due to the large number of species in the genus *Marinobacterium*, the range of 16S rRNA gene sequence similarity, DDH, AAI and ANI values between strains A346^T^ and 3-1745^T^ with other members of this genus is relatively large, and 16S rRNA gene sequence similarity values even exceed the threshold range [55] for the same genus, which may also explain the presence of *Neptuniibacter* and *Neptunomonas* strains within the *Marinobacterium* cluster in the phylogenetic tree.

### Genomic characterization and quality assessment of the genus *Marinobacterium*

The draft genome of strain A346^T^ (accession number GCA_019061305.1) was annotated with the NCBI PGAP. It contained 3351 genes (in total), including 3228 protein-coding genes and 77 RNAs genes (a complete 5S rRNA gene, a complete 16S rRNA gene, a complete 23S rRNA gene, 70 tRNA genes and four non-coding RNAs). One 16S rRNA gene sequence (1 540 bp) was detected from the genome of strain A346^T^ and shared 99.9 % similarity with the one obtained by PCR. By contrast, a total of 3086 genes were annotated in the genome of strain 3-1745^T^ (accession number GCA_013868415.1), including 2971 protein-coding genes and 79 RNA genes (three complete 5S rRNA genes, a complete 16S rRNA gene, three complete 23S rRNA genes, 68 tRNA genes and four non-coding RNAs). Only one 16S rRNA gene sequence (1 576 bp) was detected from the genome of strain 3-1745^T^ and found to have 100 % similarity with the 16S rRNA gene sequence obtained by amplification. All genomic data (excluding strains A346^T^ and 3-1745^T^) were obtained from the NCBI Genome Database (download before 20 May 2023). Detailed genomic data are shown in Table S2. The genomes of members of the genus *Marinobacterium* comprised 3.2–5.6 Mp with DNA G+C contents of 54.9–62.1 %. The DNA G+C contents of strains A346^T^ and 3-1745^T^ were respectively 58.9 and 56.4 %, occupying a medium level in the genus *Marinobacterium*. The genomic completeness and contamination were respectively above 98 % and below 3 %. Except for two strains (*Marinobacterium arenosum* CAU 1594^T^ and *Marinobacterium profundum* PAMC 27536^T^ genomes), the contig count of other bacteria was <100, and the value of N_50_ was >100 000. All this suggested that these genomic data were highly reliable. These two bacteria were not very different from other members of the genus *Marinobacterium*, which supported them belonging to this genus.

Comparative genomic analysis of the genus *Marinobacterium* was performed to determine the consistency and diversity of its members. Members of the genus have a total of 1085 core genes, which make up 20–40 % of the total (Fig. S1). The core genome is mainly distributed in amino acid metabolism, carbohydrate metabolism, energy metabolism, metabolism of cofactors and vitamins, nucleotide metabolism, overview and translation metabolism (Table S3). The accessory genome comprises to a large proportion lipid metabolism (fatty acid biosynthesis, fatty acid degradation, glycerolipid metabolism, etc), membrane transport (ABC transporters), signal transduction (two-component system), xenobiotic biodegradation and metabolism (benzoate degradation, aminobenzoate degradation, chloroalkane and chloroalkene degradation, etc), indicating that there are significant differences among members of this genus in substrate utilization, cell signal transduction and membrane transporter composition.

Gene functional classification of strains A346^T^ and 3-1745^T^ was predicted by the eggNOG database. The function classifications of strains A346^T^ and 3-1745^T^ mainly included RNA processing and modification (1, 1) (gene count of the two strains), chromatin structure and conversion (1, 1), energy production and conversion (217, 203), cell division/cycle control and chromosome partitioning (46, 45), amino acid transport and metabolism (223, 231), nucleotide transport and metabolism (72, 66), carbohydrate transport and metabolism (99, 126), coenzyme transport and metabolism (137, 137), lipid transport and metabolism (142, 103), translation, ribosomal structure and biogenesis (198, 201), transcription (189, 200), replication, recombination and repair (228, 200), cell wall/membrane/envelope biogenesis (174, 155), cell motility (121, 99), post-translational modification, protein turnover, chaperones (126, 113), inorganic ion transport and metabolism (169, 176), secondary metabolite biosynthesis, transport and catabolism (92, 73), function unknown (539, 497), signal transduction mechanisms (231, 166), intracellular trafficking, secretion, vesicular transport (106, 103), and defence mechanisms (49, 36) (Fig. S2). For example, the C_4_-dicarboxylate transporter stimulates the uptake of fumarate by bacteria [56]. Multiple ATP-binding cassette (ABC) transporters are involved in the absorption of nutrients and micronutrients [57]. In addition, pyruvate kinase, 3-phosphate dehydrogenase, fructose-1,6-bisphosphatase I and phosphoenolpyruvate carboxykinase in strains play an important role in central metabolism. The large proportion of substrate transport and metabolism genes and cell motility genes suggested that they might play a role in the growth and survival of bacteria.

### Metabolic pathway analysis of the genus *Marinobacterium*

**Fig. 5.**
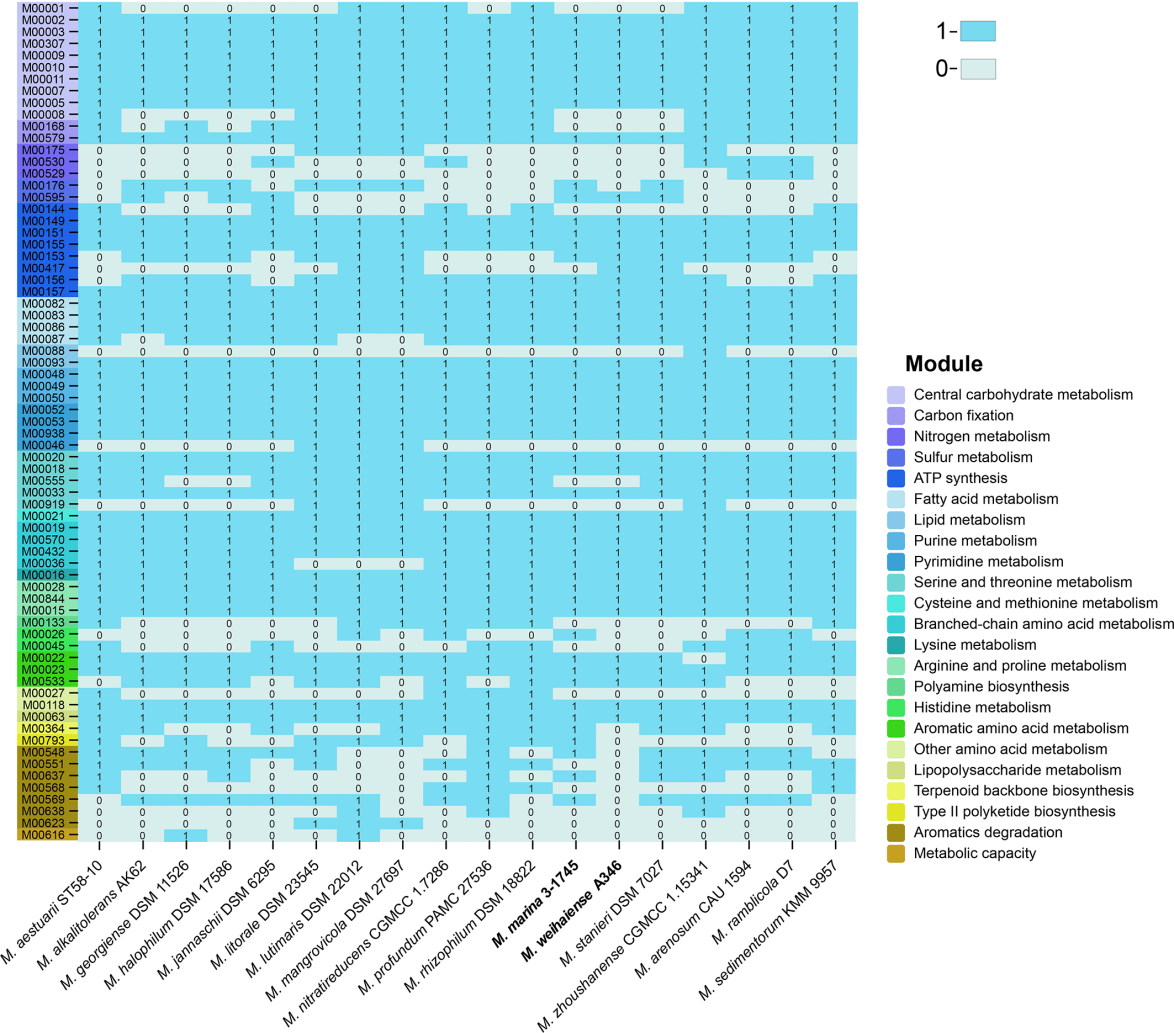
Complete and incomplete metabolism pathways of the KEGG database in the genus *Marinobacterium*: 0, incomplete metabolism pathways; 1, complete metabolism pathways. Different colour codes in the *y*-axis represent different module types.

The genomes of 18 species of the genus *Marinobacterium* were analysed. Based on functional analyses, all *Marinobacterium* strains are primarily heterotrophic, with many complete central carbohydrate metabolism, energy metabolism, lipid metabolism, nucleotide metabolism and amino acid metabolism pathways (Fig. 5). The description of metabolism pathways is given in Table S4. Nine species, including those represented by strains A346^T^ and 3-1745^T^, had the incomplete Embden–Meyerhoff pathway (M00001) and no glucokinase encoding gene was found. Central carbohydrate metabolism pathways such as gluconeogenesis, pyruvate oxidation, tricarboxylic acid (TCA) cycle, pentose phosphate pathway and PRPP (Phosphoribosyl diphosphate) biosynthesis were annotated in all bacterial genomes. However, strains A346^T^ and 3-1745^T^ were deficient in the Entner–Doudoroff pathway (M00008), which distinguishes the two novel strains from other species of the genus *Marinobacterium*. In terms of energy metabolism pathways, the nitrogen cycle is an important part of the global biogeochemical cycle, and plays an important role in biodiversity, climate change and human life [58]. Four strains had a complete nitrogen fixation pathway, indicating that they were important in providing a nitrogen source and improve oligotrophy in the marine environment. A complete pathway for dissimilatory nitrate reduction in five strains and denitrification in two strains were detected in their genomes; both consumption and utilization of nitrate make the novel strains of important potential value in improving water quality. Also, eight species including that of strain 3-1745^T^ possessed a complete assimilatory sulphate reduction pathway (M00176), and six species including those of strains A346^T^ and 3-1745^T^ had a complete thiosulphate oxidation pathway by SOX complex (M00595), indicating that the genus *Marinobacterium* may play roles in sulphur and nitrogen cycling. Experimental results for the SOX system showed that strains A346^T^, 3-1745^T^, *M. stanieri* DSM 7027^T^ and *M. maritimum* JCM 15134^T^ could oxidize thiosulphate to produce sulphate. In contrast, *M. georgiense* JCM 21667^T^ could not oxidize thiosulphate to produce sulphate, and the genome was annotated with an incomplete thiosulphate oxidation pathway. In terms of amino acid metabolism, strains A346^T^ and 3-1745^T^ had almost the same metabolic pathways, including the complete pathways for biosynthesis of leucine, threonine, cysteine and isoleucine, as well as incomplete betaine biosynthesis, ectoine degradation, polyamine biosynthesis, histidine degradation and GABA shunt. Histidine biosynthesis was annotated in the genomes of *M. lutimaris* DSM 22012^T^, *M. nitratireducens* CGMCC 1.7286^T^ and *M. marinum* 3-1745^T^, which were different from the other strains. Significantly, almost all members of the genus *Marinobacterium* possessed partly complete fatty acid biosynthesis and beta-oxidation pathways.

### PHA production potential of the genus *Marinobacterium*

PHAs are microbial intracellular metabolites with plastic-like material properties, biodegradable properties and broad application prospects. PHAs are always produced under imbalanced metabolic conditions [59]. PHAs in bacteria serve as carbon and energy reserves that help strains to resist adverse stress conditions [60, 61]. As far as we are aware, three species of the genus *Marinobacterium* (*M. nitratireducens*, *M. sediminicola* and *M. zhoushanense*) have the ability to produce PHAs [17].

**Fig. 6.**
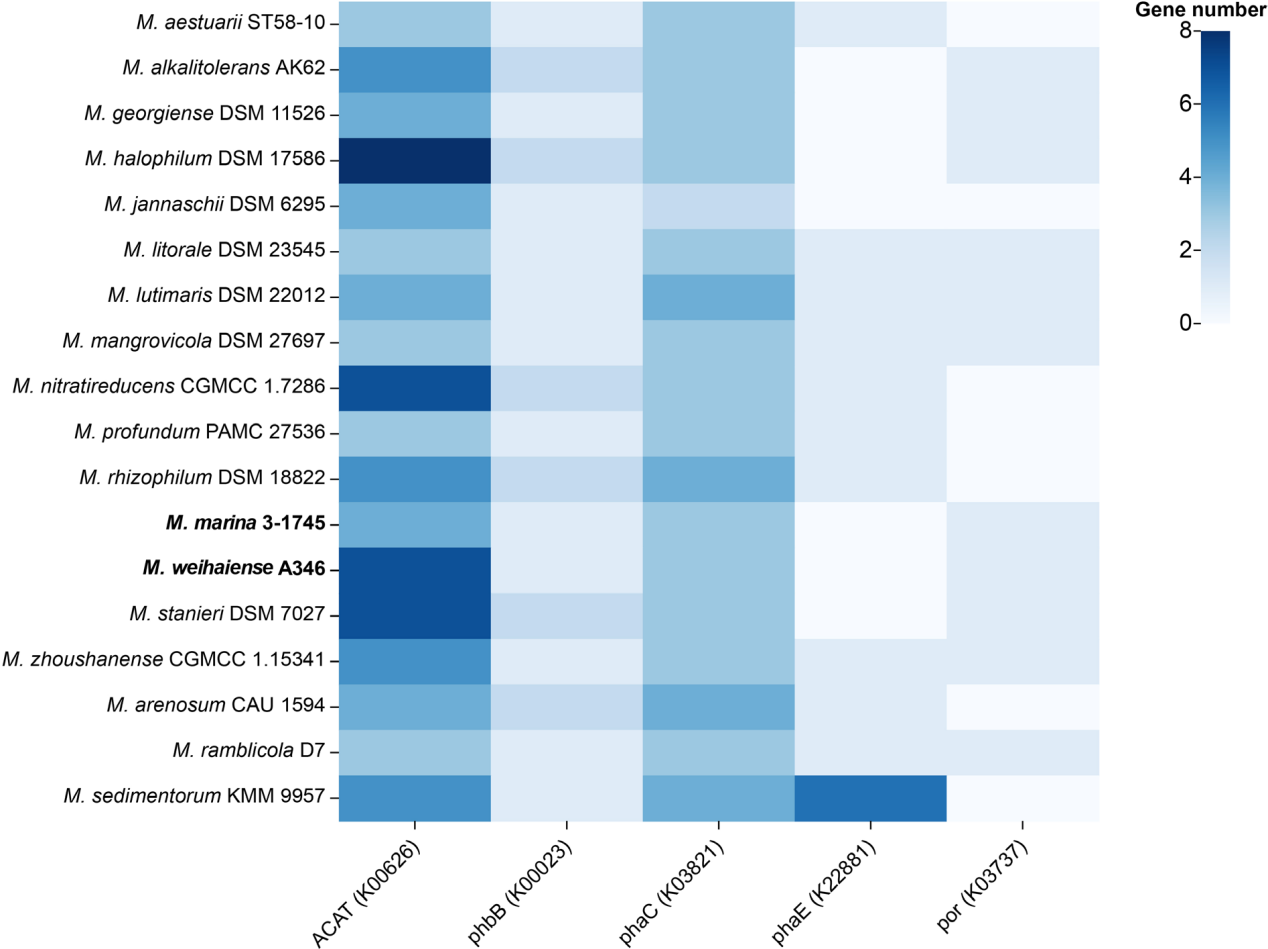
PHA synthase genes in *Marinobacterium* genomes. Numbers represent the gene count. K00626: *ACAT*, *atoB*; acetyl-CoA C-acetyltransferase; K00023: *phbB*; acetoacetyl-CoA reductase; K03821: *phaC*, *phbC*; poly[(*R*)−3-hydroxyalkanoate] polymerase subunit PhaC; K22881: *phaE*; poly[(*R*)-3-hydroxyalkanoate] polymerase subunit PhaE; K03737: *por*, *nifJ*; pyruvate-ferredoxin/flavodoxin oxidoreductase.

**Fig. 7.**
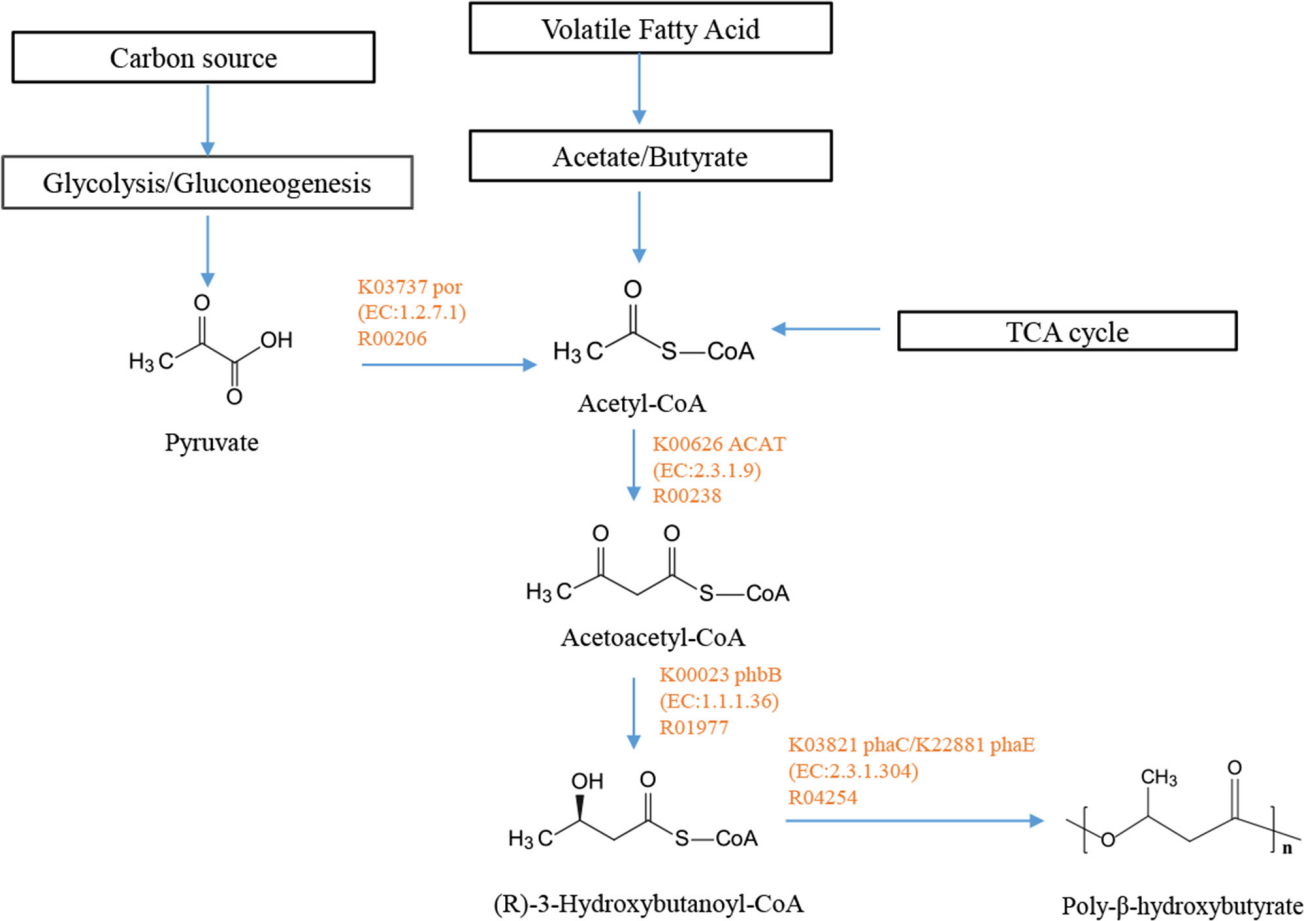
PHA synthetic pathways in the genus *Marinobacterium*. The enzymes shown in yellow are present in strains A346^T^ and 3-1745^T^.

PhaA, PhaB and PhaC for the biosynthesis of PHAs were prevalent in the genus *Marinobacterium*. All genomes contained multiple PHA synthases (Fig. 6). Strains A346^T^ and 3-1745^T^ both possessed three PHA synthase (*phaC*) genes and one acetoacetyl-CoA reductase (*phaB*) gene. In particular, strain A346^T^ encoded up to seven acetyl-CoA C-acetyltransferase (*phaA*) genes, compared to up to four *phaA* genes for strain 3-1745^T^. PHA production was accomplished through three steps sequentially mediated by acetyl-CoA C-acetyltransferase (EC 2.3.1.9; *ACAT*), acetoacetyl-CoA re-ductase (EC 1.1.1.36; *phaB*) and polyhydroxyalkanoate synthase (EC 2.3.1.304; *phaC*) or poly[(*R*)-3-hydroxyalkanoate] polymerase subunit PhaE (*phaE*). Micro-organisms are able to use different carbon sources such as sugars and fatty acids to form acetyl-CoA through multiple metabolic pathways, by acetyl-CoA C-acetyltransferase (*phaA*) to produce acetoacetyl-CoA. Acetoacetyl-CoA was reduced to (*R*)-3-hydroxybutanoyl-CoA by acetoacetyl-CoA reductase (*phaB*), and then (*R*)-3-hydroxybutanoyl-CoA was reduced to poly-β-hydroxybutyrate by PHA synthase (*phaC*). Specific complete metabolic pathways are shown in Fig. 7. All strains possessed the complete beta-oxidation pathway (M00086), producing acetyl-CoA. These results indicated that these 15 species, including those represented by the two novel strains A346^T^ and 3-1745^T^, of the genus *Marinobacterium* might synthesize PHAs.

### Prediction of biosynthetic gene clusters

**Fig. 8.**
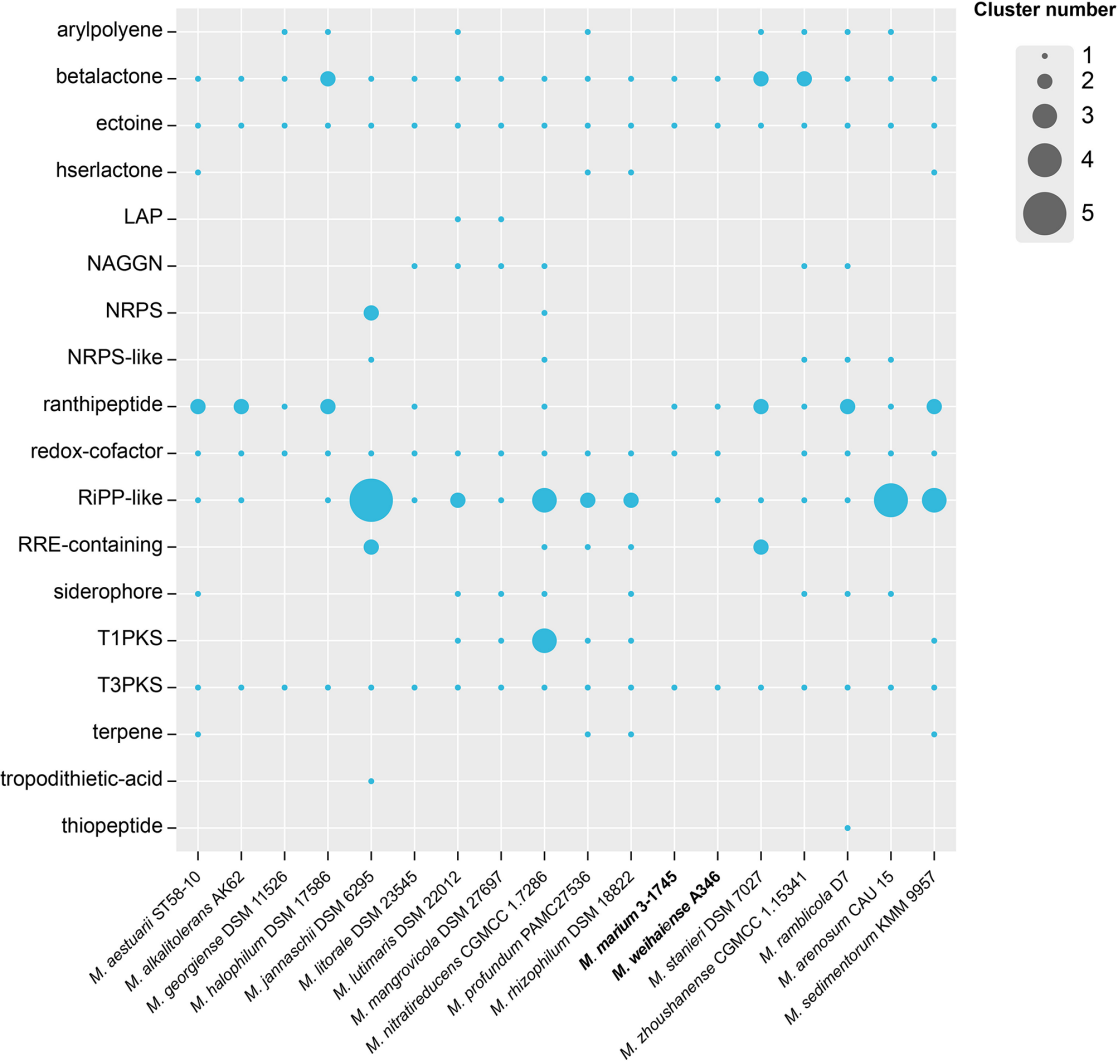
Prediction of biosynthetic gene clusters for *Marinobacterium* strains annotated by the antiSMASH database. Bubble sizes represent the number of gene clusters.

In the genomes of the 18 type strains of the genus *Marinobacterium*, potential production of secondary metabolites was annotated by antiSMASH. All results are showed in Fig. 8. The results of genome analysis revealed the presence of multiple biosynthetic gene clusters (BGCs), including arylpolyene, betalactone, ectoine, hserlactone (homoserine lactone), LAP (Linear azol(in)e-containing peptides), NAGGN (nacetyl glutaminyl glutamine amide), NRPS (non-ribosomal peptide synthetases), ranthipeptide, redox-cofactor, RiPPs (ribosomally synthesised and post-translationally modified peptide product), RRE-containing (containing -RiPP recognition element-containing cluster), siderophore, T1PKS (polyketide synthases), T3PKS, terpene and tropodithietic acid. All genomes contained some common BGCs (betalactone, ectoine and T3PKS). The gene cluster for ectoine biosynthesis probably underlies the salinity tolerance strategy. Strain 3-1745^T^ had gene clusters for the synthesis of five products, including ectoine, redox-cofactor, ranthipeptide, betalactone and T3PKS gene cluster. Strain A346^T^ contained six gene clusters for the synthesis of ectoine, redox-cofactor, ranthipeptide, beta-lactone, RiPP-like and T3PKS. As all species of the genus *Marinobacterium* live in saline environments, the synthesis of multiple BGCS may be an adaptation to such environments [62]. *M. jannaschii* DSM 6295^T^ and *M. nitratireducens* CGMCC 1.7286^T^ had the largest number of gene clusters (15) among all strains. Members of the genus *Marinobacterium* isolated from scleractinian corals can produce marinoquinolones and marinobactoic acid that have antimicrobial activity and cytotoxicity [15]. These results could be utilized to support future microbial prospecting of the genus *Marinobacterium*.

In summary, strains A346^T^ and 3-1745^T^ carried some genes encoding compatible solute production (*proA*, *proB*, *proC*, *proQ*) and different genes encoding compatible solute transport as well as the presence of multiple BGCs including ectoine, and can survive in highly saline environments.

### Resistome analyses

The results obtained in the antimicrobial susceptibility tests showed that strains 3-1745^T^ and A346^T^ were resistant to lincomycin (2 µg), vancomycin (30 µg), kanamycin (30 µg), streptomycin (10 µg), neomycin (30 µg) and tobramycin (10 µg), but susceptible to ofloxacin (5 µg), ampicillin (10 µg), penicillin (10 µg), polymyxin B (300 µg), ceftriaxone (30 µg), chloramphenicol (30 µg) and cefotaxime sodium (30 µg). There were some differences between the two strains. Strain A346^T^ was resistant to carbenicillin (100 µg), norfloxacin (30 µg), erythromycin (15 µg), clarithromycin (10 µg), rifampin (5 µg), tetracycline (30 µg) and gentamycin (10 µg), but strain 3-1745^T^ was susceptible to these compounds. The genomes of strains 3-1745^T^ and A346^T^ had the genes *AAC (3)-IIa*, *CrcB*, *mexY*, *npmA* and *rmtB* that are resistant to aminoglycoside antibiotics, which may explain their resistance to tobramycin and streptomycin. This may also be the cause of the resistance of strain A346^T^ to gentamicin. Mobile genetic elements with replication/recombination and integration/excision/repair functions have been annotated around these genes, indicating that they have horizontal transfer risk [63–65]. In both strains 3-1745^T^ and A346^T^, there are vancomycin resistance (*van*) gene clusters, *tet (O*), *adeb* and *HelR*, which may explain their resistance to vancomycin, tetracycline and rifampicin. Mobile genetic elements with replication/recombination and integration/excision/repair functions are also annotated around these genes, suggesting that they may have horizontal transfer risk [63–65]. Genome analyses of strains 3-1745^T^ and A346^T^ showed that they contained the resistance-nodulation-cell division (RND) antibiotic efflux pump gene family, multiple antimicrobial extrusion protein (Na^+^/drug antiporter) and MATE family of MDR efflux pumps. This suggested that resistance in these bacteria might be associated with resistance genes. Strain 3-1745^T^ was resistant to multiple classes of antibiotics (lincosamides, aminoglycosides and glycopeptides), and strain A346^T^ was resistant to lincosamides, ansamycins, macrolides, quinolones, aminoglycosides and glycopeptides (among others). Due to the multi-resistance characteristics of these two strains [66], this may also provide a new way to add antibiotics in industrial production to prevent contamination by other bacteria.

### Microbial substrate preference

**Fig. 9.**
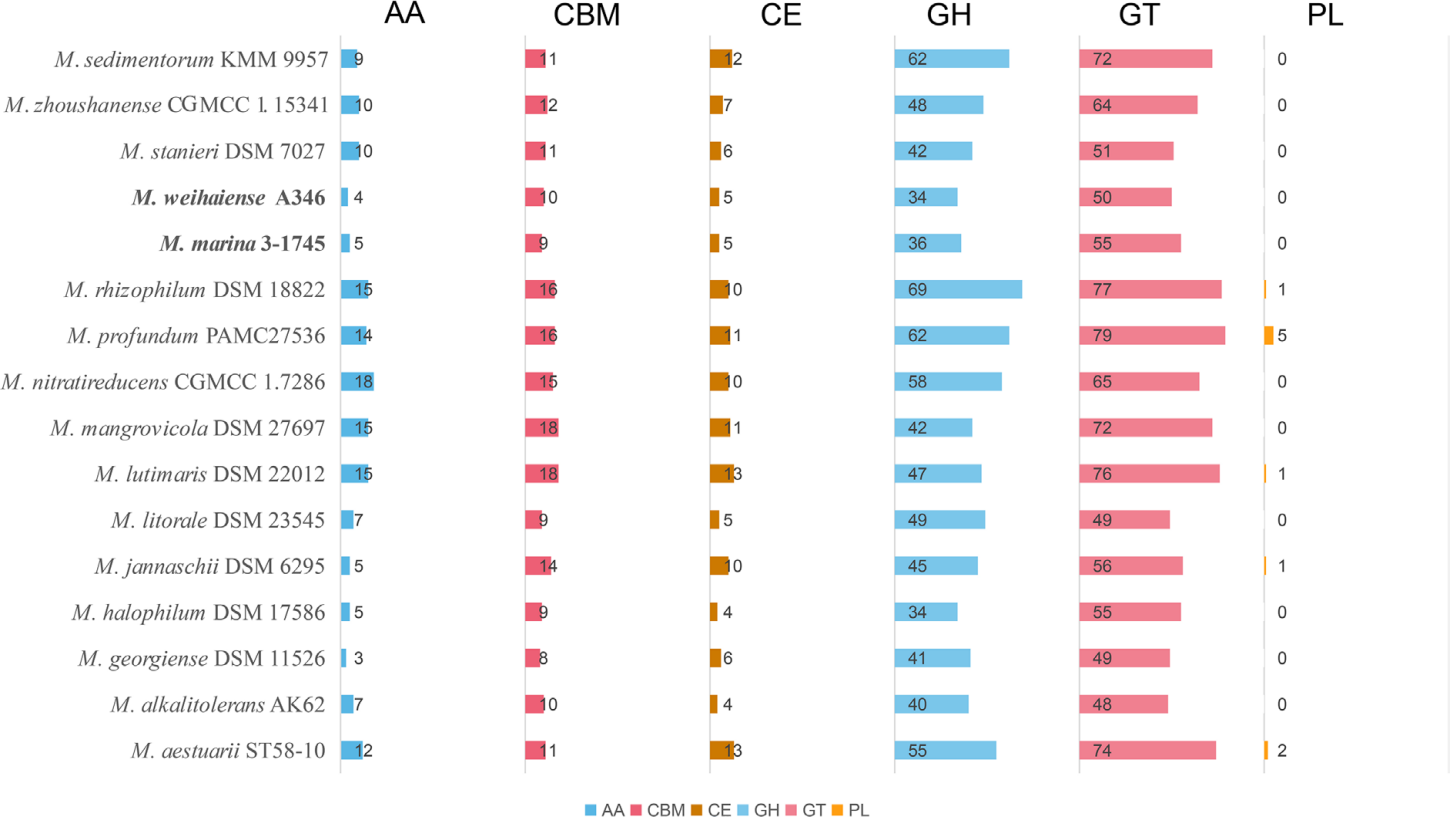
Comparison of carbohydrate-active enzymes (CAZymes) between various *Marinobacterium* strains. AA: auxiliary activities; CBM: carbohydrate-binding module; CE: carbohydrate esterases; GH: glycoside hydrolases; GT: glycosyltransferases; PL: polysaccharide lyases.

**Fig. 10.**
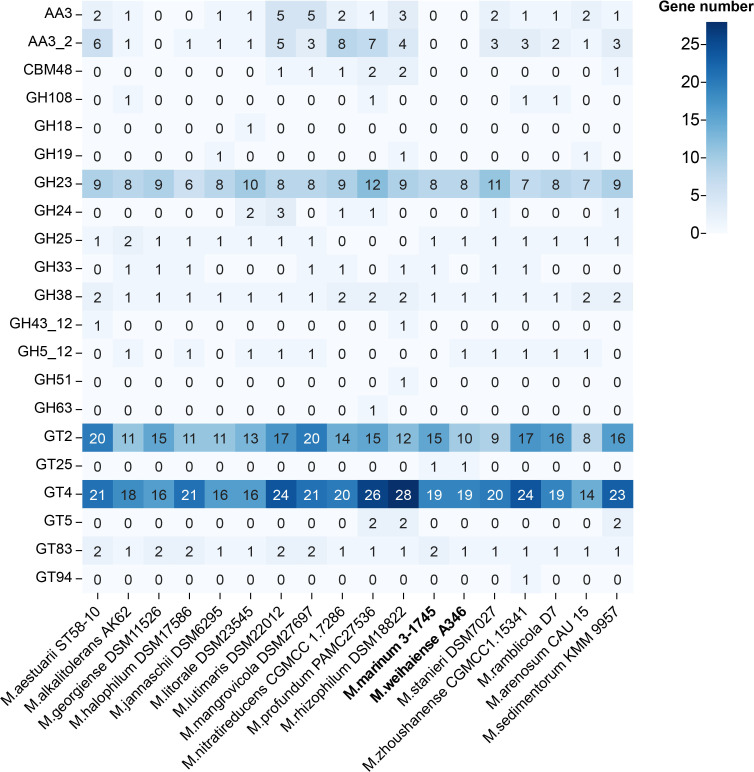
Predicted number of carbohydrate-active enzymes detected in the genus *Marinobacterium*. The shade of the colour represents the number of genes.

To investigate if the genus *Marinobacterium* could utilize oligosaccharides and polysaccharides, the composition of carbohydrate-active enzymes (CAZymes) was compared via the Carbohydrate-Active Enzymes Database. The results of genome analysis revealed that all strains had analogous CAZymes compositions, and glycosyltransferase (GTs) and glycoside hydrolase (GHs) were their dominant CAZymes (Figs 9 and 10). The description of CAZymes families is shown in Table S5. Polysaccharide lyases were absent in almost all strains. Strains A346^T^ and 3-1745^T^ contained 110 and 103 CAZymes, respectively. Glycoside hydrolase families GH23, GH24, GH25 and GH108 potentially involved in lysozyme production were detected, which could reflect a certain bacteriostatic ability of these strains, with possible application in food preservation and preservation, medicine and biological engineering. The other CAZyme families related to degradation of xylan (GH38, GH43_12 and GH51), cellobiose (GH5_12, GT94) and other multiple oligosaccharides (AA3, GT2 and GT4) were annotated in some strains. These results suggest that the genus *Marinobacterium* mainly utilizes oligosaccharides, consistent with carbon utilization results for strains A346^T^, 3-1745^T^, *M. stanieri* DSM 7027^T^, *M. maritimum* JCM 15134^T^ and *M. georgiense* JCM 21667^T^ (Tables 1 and S6).

**Table 1.** Differential phenotypic characteristics between strains A346^T^, 3-1745^T^ and related *Marinobacterium* type strains Strains: 1, A346^T^; 2, 3-1745^T^; 3, *M. georgiense* JCM 21667^T^; 4, *M.stanieri* DSM 7027^T^; 5, *M. maritimum* JCM 15134^T^. +, Growth; w, weak growth; −, no growth. All data were obtained in this study unless indicated otherwise.

Characteristic	1	2	3	4	5
Temperature for growth (°C)					
Range	4–44	4–40	4–41*	10–37†	7–37‡
Optimum	30–37	37	37*	25–30†	25–28‡
Cell size (μm)	0.6–0.7×1.2–2.3	0.4–0.5×1.1–1.7	0.5–0.7×1.6–2.3*	0.6–0.8×1.3–2.2†	0.5–0.6×0.8–0.9‡
Salinity growth range (%)	0.5–18	0–12	0–12*	0.5–8†	0.5–7‡
Optimum NaCl concentration (%, w/v)	1	0.5	0.5–2*	2†	1–2‡
pH growth range	6.0–10.0	6.0–9.5	5.0–10.0*	5.0–12.0†	5.5–9.0‡
Optimum pH growth	7.0	7.0	7.0–8.0*	7.0†	7.5–8.0‡
Motility	+	+	+*	+†	+‡
Nitrate reduction	–	–	–*	–†	–‡
Hydrolysis of:					
Tween 20	–	+	–	–	–
Tween 40	–	+	–	+	–
Tween 60	–	–	–	–	–
Tween 80	–	–	+∗	–	–‡
Citrate utilization	–	–	w	+	+
Enzymatic activity					
Oxidase	+	+	+*	+†	+‡
Catalase	+	–	+*	+	+‡
Acid phosphatase	+	–	+	+	+
Acid production from:					
l-Arabinose	+	–	–	–	–
Ribose	–	+	+	+	+
l-Xylose	+	–	–	–	–
Oxidation of:					
Saccharic acid	+	–	–	–	–
Malic acid	–	w	w	–	+
DNA G+C content (%)	58.9	56.4	54.9*	55.6†	57.9‡

*Data from [1].

†Data from [74].

‡Data from [75].

In addition, to explore the ability of this genus to utilize other substrates, annotated analysis of the genome revealed the existence of a variety of esterases (esterase, acetyl esterase and pimeloyl-[acyl-carrier protein] methyl ester esterase) and enzymes (acetate-CoA synthase, acetate kinase and phosphate acetyltransferase) related to acetic acid metabolism. It has also been confirmed experimentally that strains A346^T^, 3-1745^T^, *M. stanieri* DSM 7027^T^, *M. maritimum* JCM 15134^T^ and *M. georgiense* JCM 21667^T^ were positive for activity of alkaline phosphatase, esterase (C4), esterase lipase (C8) and lipase (C14) and positive for the utilization of l-lactic acid, α-hydroxy-butyric acid, β-hydroxy-d, l-butyric acid, propionic acid, acetic acid, sodium bromate, sodium butyrate, etc. In addition, strain 3-1745^T^ was positive for hydrolysis of Tweens 20 and 40. It is reported that several species from the genus *Marinobacterium* including *M. nitratireducens*, *M. sediminicola*, and *M. zhoushanense* can producePHAs using sugars and volatile fatty acids as the carbon source [24].

### Flagellar motor capacity

**Fig. 11.**
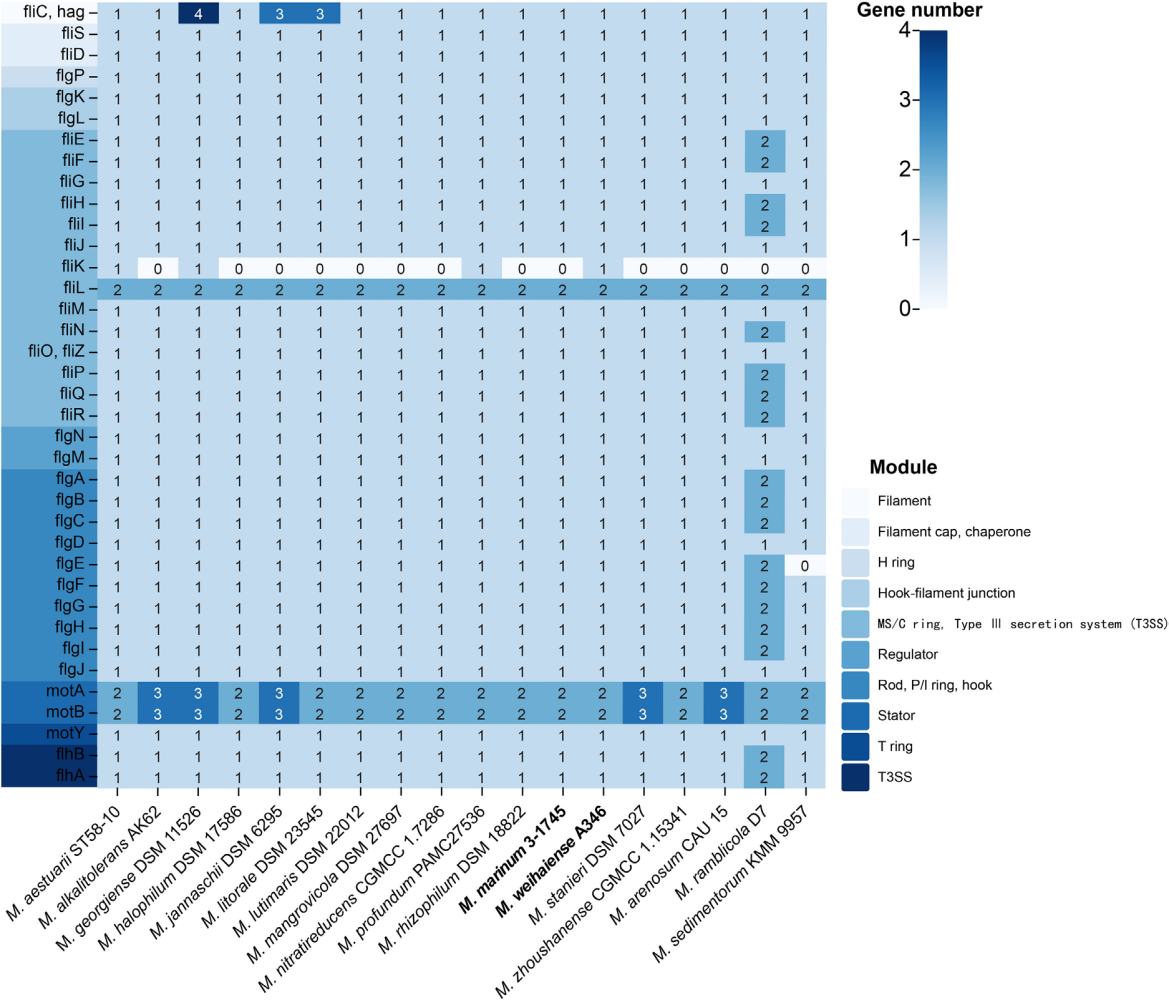
Flagella synthase genes in *Marinobacterium* sp. genomes. Numbers represent the gene count.

Flagellar movement plays an active role in many biological functions of bacteria, such as the formation of bacteria–host symbiosis, pathogenicity and antibiotic resistance [67, 68]. Through scanning electron microscopy and transmission electron microscopy, cell morphology showed that cells of strains A346^T^ and 3-1745^T^ were both rod-shaped with a single polar flagellum and approximately 0.6–0.7 µm wide and 1.2–2.3 µm long for strain A346^T^ and approximately 0.4–0.5 µm wide and 1.1–1.7 µm long for strain 3-1745^T^ (Table 1 and Fig. S3). Colony sizes for strains A346^T^ and 3-1745^T^ were 0.2–1.2 and 0.1–2.0 mm, respectively. Genome analysis revealed that each bacterium in the genus *Marinobacterium* contained multiple genes that encode flagella, which were used to synthesize its components (filament, filament cap, H ring, hook-filament junction, MS/C ring, type III secretion system, P/L ring and T ring Stator, etc.) [69, 70] (Fig. 11). Bacteria may make directional responses to environmental factors of different gradients through flagellar movement, so as to favour stimuli and avoid harmful stimuli [71]. Cells of members of the genus *Marinobacterium* were motile by a single or two polar flagella [72]. In the same family, the discovery of thick, polar flagellar filaments in *Oleibacter marinus* has expanded the known diversity of flagellar architecture [73]. The results suggested that flagella are a common feature of the genus, which could help bacteria actively gravitate toward nutrients or avoid harmful chemicals.

### Physiological and chemotaxonomic analyses

Strain A346^T^ could grow at 4–44 °C (optimum, 30–37 °C), with 0.5–18 % (w/v) NaCl (optimum, 1%) and at pH 6.0–10.0 (optimum, pH 7.0). Strain 3-1745^T^ could grow at 4–40 °C (optimum, 37 °C), with 0–12 % (w/v) NaCl (optimum, 0.5 %) and at pH 6.0–9.5 (optimum, pH 7.0) (Table 1). By analysing the genome of the strains, A346^T^ and 3-1745^T^ contained multiple salt-tolerance genes (*trkA*, *trkH*, *proA*, *proB*, *proC* and *proQ*), which may be related to a wide salt tolerance shown by both strains during rearing in the lab.

The dominant fatty acids (>5 %) of strain A346^T^ were summed feature 3 (C_16 : 1_ω7*c*/C_16 : 1_ω6*c*) (29.2 %), C_16 : 0_ (24.8 %), summed feature 8 (C_18 : 1_ω7*c*/C_18 : 1_ω6*c*) (22.0 %), C_12 : 0_ (6.4 %) and C_10 : 0_ 3-OH (5.9 %). The dominant fatty acids (>5 %) of strain 3-1745^T^ were summed feature 8 (C_18 : 1_ω7*c*/C_18 : 1_ω6*c*) (31.0 %), summed feature 3 (C_16 : 1_ω7*c*/C_16 : 1_ω6*c*) (27.9 %) and C_16 : 0_ (27.1 %). The cellular fatty acid compositions of A346^T^ and 3-1745^T^ and related type strains are shown in Table 2. Some dominant fatty acids of strains A346^T^ and 3-1745^T^ were similar with those of the other related type strains, such as C_16 : 0_ and C_10 : 0_ 3-OH, but there were differences in the proportions of some fatty acids (C_17 : 0_ cyclo, C_14 : 0_). In strain A346^T^, the contents of C_12 : 0_, C_14 : 0_ and C_10 : 0_ 3-OH were greater than those of the other four strains. The sole respiratory quinone of both strains 3-1745^T^ and A346^T^ was ubiquinone-8. The major polar lipids of strain A346^T^ were phosphatidylglycerol (PG), phosphatidyl-ethanolamine (PE), an unidentified amino lipid (AL) and three unidentified lipids (L). The major polar lipids of strain 3-1745^T^ were phosphatidylglycerol (PG), phosphatidyl-ethanolamine (PE), diphosphatidylglycerol (DPG), an unidentified aminolipid (AL), an unidentified phospholipid and five unidentifified lipids (L) (Fig. S4). The polar lipids of strains A346^T^, 3-1745^T^ and related type strains had some in common (PE, PG), and strains A346^T^ and 3-1745^T^ had an unidentified aminolipid (AL) which was different from the other strains. In addition, strain 3-1745^T^ contained one unidentified phospholipid (PL) and five unidentifified lipids, which were not found in the other strains.

**Table 2.** Cellular fatty acid composition (%) of strains A346^T^, 3-1745^T^ and related *Marinobacterium* type strains Strains: 1, A346^T^; 2, 3-1745^T^; 3, *M. georgiense* JCM 21667^T^; 4, *M. stanieri* DSM 7027^T^; 5, *M. maritimum* JCM 15134^T^. All data were obtained in this study. Fatty acids present at >5 % are marked in bold. −, Not detected; tr, trace (<1 %).

Fatty acid	1	2	3	4	5
Straight-chain fatty acids					
C_10 : 0_	4.2	2.4	3.8	tr	2.7
C_12 : 0_	**6.4**	2.6	1.9	4.3	2.9
C_14 : 0_	1.1	tr	tr	tr	tr
C_16 : 0_	**24.8**	**27.1**	**30.6**	**22.9**	**24.4**
C_18 : 0_	tr	1.3	1.2	2.0	tr
Cyclopropane acids					
C_17 : 0_ cyclo	–	–	–	–	3.4
Hydroxy fatty acids					
C_10 : 0_ 3-OH	**6.0**	4.3	4.3	4.4	4.5
Summed features*					
3	**29.17**	**27.93**	**19.05**	**21.13**	**25.26**
8	**22.0**	**31.0**	**36.5**	**40.5**	**32.7**

*Summed features represent groups of two or three fatty acids that could not be separated by GLC with the MIDI system. Summed feature 3 consisted of C_16 : 1_ω7*c*/C_16 : 1_ω6*c* and summed feature 8 consisted of C_18 : 1_ω7*c*/C_18 : 1_ω6*c*.

## Description of *Marinobacterium weihaiense* sp. nov.

*Marinobacterium weihaiense* (wei.hai.en’se. N.L. neut. n. *weihaiense* originating from Weihai, China).

Cells are Gram-stain-negative, facultatively anaerobic, rod-shaped, motile and almost 0.6–0.7 µm width and 1.2–2.3 µm length. Colony size is 0.2–1.2 mm. Colonies are circular at 35 °C on MA after 2 days. Grows at 4–44 °C (optimum, 30–37 °C), with 0.5–18 % (w/v) NaCl (optimum, 1%) and at pH 6.0–10.0 (optimum, pH 7.0). Positive for Voges–Proskauer reaction. The activity of oxidase and catalase, as well as gelatinase, alkaline phosphatase, esterase (C4), leucine arylamidase, acid phosphatase and naphthol-AS-BI-phosphohydrolase are positive. The activity of esterase lipase (C8), lipase (C14), cystine arylamidase, *N*-acetyl-β-glucosaminidase and valine arylamidase are weakly positive. Negative for hydrolysis of agar, alginate, CM-cellulose, starch, Tweens 20, 40, 60 and 80, DNA and casein. Positive for utilization of tagatose, potassium 2-ketogluconate, potassium 5-ketogluconate, aesculin ferric citrate, amygdalin, methyl-β-d-xylopyranoside, l-xylose, l-arabinose, cellobiose, mannitol, galactose, l-rhamnose, fusidic acid, serine, l-alanine, glucuronamide, saccharic acid, α-ketoglutaric acid and acetic acid. The major fatty acids are summed feature 3 (C_16 : 1_ω7*c*/C_16 : 1_ω6*c*), C_16 : 0_, summed feature 8 (C_18 : 1_ω7*c*/C_18 : 1_ω6*c*) and C_12 : 0_. The sole respiratory quinone is ubiquinone-8. The major polar lipids are phosphatidylglycerol, phosphatidylethanolamine, an unidentified amino lipid and three unidentifified lipids. The genomic DNA G+C content of the type strain is 58.9 %.

The type strain, A346^T^ (=KCTC 92007^T^=MCCC 1H00492^T^=SDUM032111^T^), was isolated from marine sediment from the coast of Weihai, China (36° 58′ 37″ N 122° 2 ′37″ E). The GenBank accession number for the 16S rRNA gene sequence of strain A346^T^ is MZ434947.1 and the draft genome has been deposited in GenBank under accession number JAHQZT000000000.1.

## Description of *Marinobacterium marinum* sp. nov.

*Marinobacterium marinum* (ma.ri’num. L. neut. adj. *marinum* of or belonging to the sea, marine).

Cells are rod-shaped, Gram-stain-negative, facultatively anaerobic, approximately 0.4–0.5 µm width and 1.1–1.7 µm length, and motile by means of a single polar flagellum. Colony size is 0.1–2.0 mm. Grows at 4–40 °C (optimum, 37 °C), with 0–12 % (w/v) NaCl (optimum, 0.5 %) and at pH 6.0–9.5 (optimum, pH 7.0). Positive results in tests for oxidase activity, Voges–Proskauer reaction and hydrolysis of Tweens 20 and 40, but negative for catalase activity, nitrate reduction, hydrolysis of agar, alginate, CM-cellulose, starch, Tweens 60 and 80, DNA and casein. Positive for activity of gelatinase, alkaline phosphatase, esterase (C4), leucine arylamidase and naphthol-AS-BI-phosphohydrolase and positive for utilization of glycerol, d-ribose, l-rhamnose, aesculin ferric citrate, cellobiose, raffinose, tagatose, potassium 2-ketogluconate, potassium 5-ketogluconate, fucose, fusidic acid, serine, l-alanine, l-glutamic acid, l-pyroglutamic acid, galacturonic acid, glucuronic acid, glucuronamide, l-lactic acid, α-ketoglutaric acid, propionic acid and acetic acid. The activity of esterase lipase (C8), lipase (C14) and valine arylamidase are weakly positive. The major fatty acids are summed feature 8 (C_18 : 1_ω7*c*/C_18 : 1_ω6*c*), summed feature 3 (C_16 : 1_ω7*c*/C_16 : 1_ω6*c*) and C_16 : 0_. The major polar lipids are phosphatidylglycerol, phosphatidylethanolamine, diphosphatidylglycerol, an unidentified amino lipid, an unidentified phospholipid and five unidentified lipids. The sole respiratory quinone is ubiquinone-8. The genomic DNA G+C content of the type strain is 56.4 %.

The type strain, 3-1745^T^ (=KCTC 72925^T^=MCCC 1H00422^T^=SDUM 032109^T^), was isolated from marine sediment from the coast of Weihai, China (36° 58′ 37″ N 122° 2′ 37″ E). The GenBank accession number for 16S rRNA gene sequence of strain 3-1745^T^ is MW391814.1 and the draft genome has been deposited in GenBank with accession number JACEMT000000000.1.

## Conclusions

In this study, strains A346^T^ and 3-1745^T^ were isolated from coastal sediment taken from China. They were affiliated to the genus *Marinobacterium*, and represented the novel species *Marinobacterium weihaiense* and *Marinobacterium marinum*, respectively. Strains A346^T^ and 3-1745^T^ carried multiple genes encoding compatible solute production and compatible solute transport to help them resist the high-salinity environment. Strain 3-1745^T^ had five BGCs, including ectoine, redox-cofactor, ranthipeptide, betalactone and T3PKS. Strain A346^T^ contained six BGCs for synthesis of ectoine, redox-cofactor, ranthipeptide, beta-lactone, RiPP-like and T3PKS.

Genomic analyses of the two new strains indicated they have the capacity to utilize lipids and volatile fatty acids, and can grow rapidly in a high-salinity environment and have a strong ability to adapt to the environment. Through genomic analysis of the genus *Marinobacterium*, members of this group have a complete PHA synthesis pathway. This indicates that some members of the genus *Marinobacterium* have the ability to oxidize thiosulphate to sulphate. Some members had a complete nitrogen fixation, dissimilatory nitrate reduction and denitrification pathway, indicating that they may be important in providing a nitrogen source, improve oligotrophic marine environments and improve water quality. In this study, the isolation and identification of strains A346^T^ and 3-1745^T^ has further enriched the marine microbial resources and gene pool particularly known for this bacterial genus which has been poorly exploited from a biotechnological standpoint. There are multiple metabolic pathways in strains A346^T^ and 3-1745^T^, including carbohydrate metabolism, energy metabolism, lipid metabolism, nucleotide metabolism and amino acid metabolism, which play an important role in the global carbon, nitrogen and sulphur cycle and are of great significance for maintaining global ecological balance. They have the potential ability to synthesize PHAs and multiple secondary metabolites, and have broad application prospects in the materials, agriculture, food and biomedical fields.

## Supplementary Data

Supplementary material 1
