# Supplementary material for: Phenotypic and genotypic characterization of Marinobacterium weihaiense sp. nov. and Marinobacterium marinum sp. nov., isolated from marine sediment, and genomic properties of the genus Marinobacterium
Source: Microb Genom. 2024 Jan 24;10(1):001182. doi: 10.1099/mgen.0.001182 (PMC10868613; doi:10.1099/mgen.0.001182)
Supplement: Supplementary material 1 [file mgen-10-1182-s001.pdf]

Supplementary materials

Phenotypic and genotypic characterization of *Marinobacterium weihaiense* sp. nov. and *Marinobacterium marinum* sp. nov., isolated from marine sediment, and genomic properties of the genus *Marinobacterium*

Xin-Jiang Liu[1,2], Ke-Lei Zhu[2], Yu-Qi Ye[2], Ze-Tian Han[2], Xin-Yun Tan[2], Zong-Jun Du[2,3]*, Meng-Qi Ye[1,2,3] *

[1]Shenzhen Research Institute of Shandong University, Shenzhen, Guangdong, 518057, PR China.

[2] Marine College, Shandong University, Weihai, Shandong, 264209, PR China.

[3] Weihai Research Institute of Industrial Technology of Shandong University, Weihai, 264209, PR China.

***Correspondence**: Meng-Qi Ye, Email: yemengqi@sdu.edu.cn

Zong-Jun Du, Email: duzongjun@sdu.edu.cn

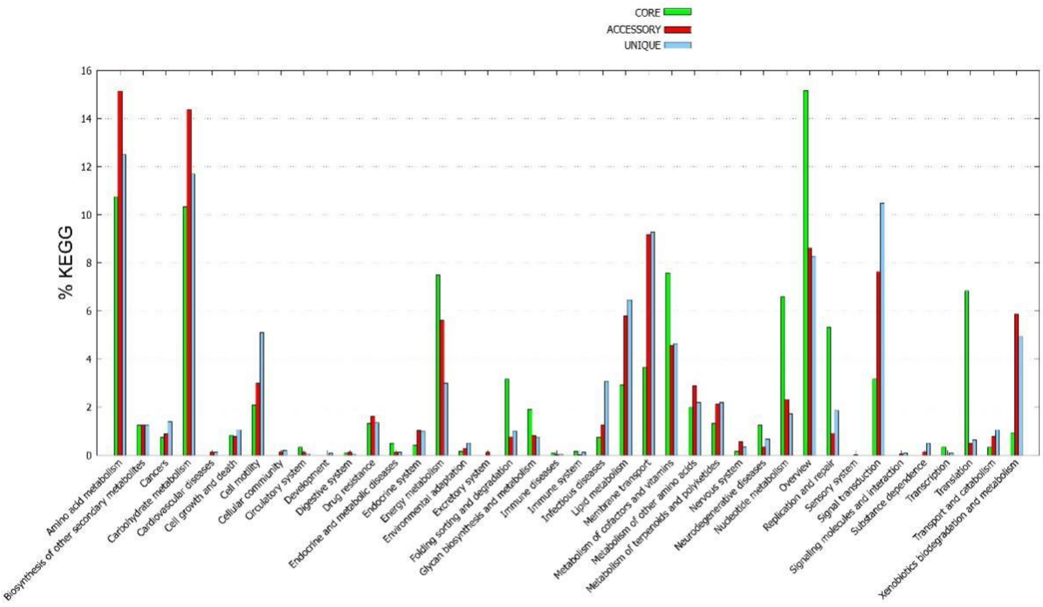

Supplementary Figure 1. The distribution of core genes, accessory genes and unique genes to different metabolic pathways in the genus *Marinobacterium*. green: core genes; red: accessory genes; blue: unique genes.

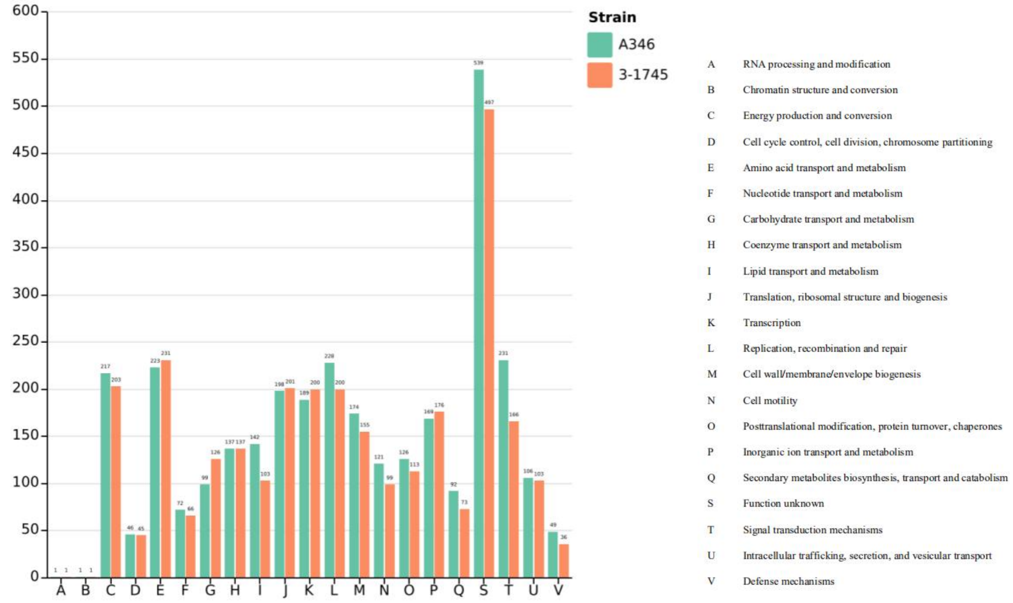

Supplementary Figure 2. Compare with clusters of orthologous groups (COGs) between strains A346^{T} and 3-1745^{T}. x-axis: gene functional categories; y-axis: gene numbers.

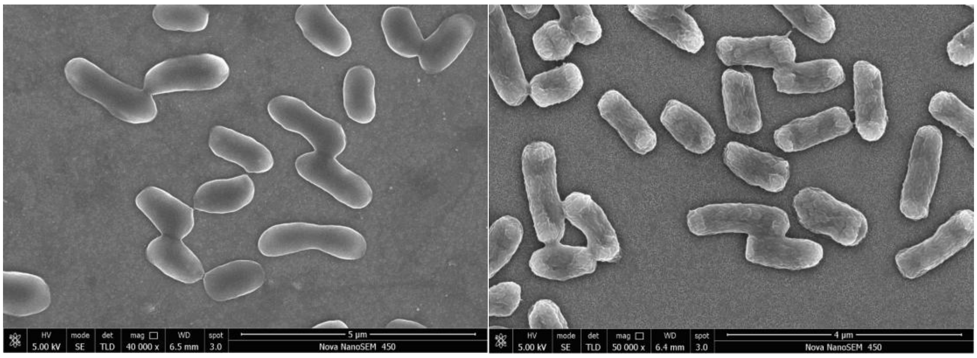

A B

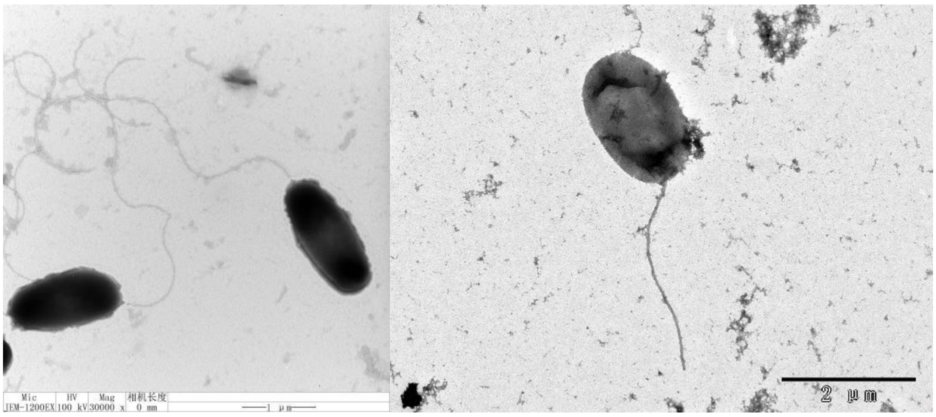

C D

Supplementary Figure 3. **(A)** Cell morphology of strain A346^{T} shown by scanning electron microscopy.Scale bar, 5 μm. **(B)** Cell morphology of strain 3-1745^{T} shown by scanning electron microscopy.Scale bar, 4 μm. **(C)** Cell morphology of strain 3-1745^{T} shown by transmission electron microscopy.Scale bar, 1μm. (**D**) Cell morphology of strain A346^{T} shown by transmission electron microscopy.Scale bar, 2μm.

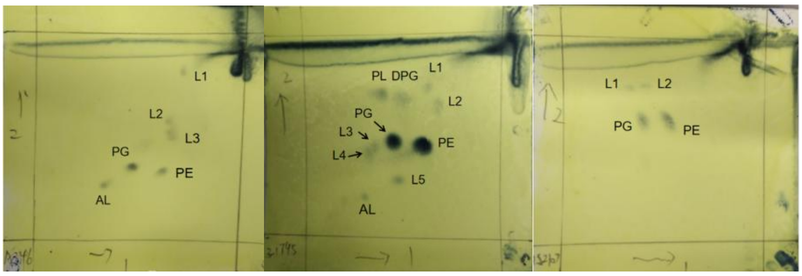

1 2 3

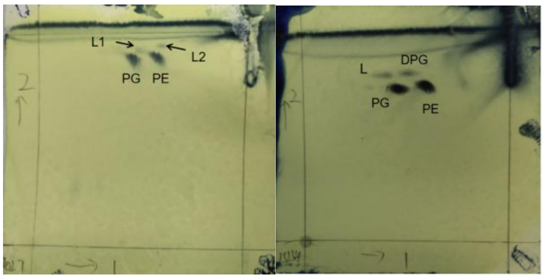

4 5

Supplementary Figure 4. Two-dimensional TLC of polar lipids of five strains. The plate was sprayed with 5% phosphomolybdic acid and heated at 150 °C to show all lipids. **1, A346^{T}; 2, 3-1745^{T}**; 3, *M. georgiense* JCM 21667^{T}; 4, *M.stanieri* DSM 7027^{T}; 5, *M. maritimum* JCM 15134^{T}. PG: phosphatidylglycerol, PE: phosphatidylethanolamine, DPG: diphosphatidylglycerol, AL: unidentified aminolipid, PL: unidentified phospholipid, L: unidentifified lipids.

Supplementary Table 1. The 16S rRNA gene sequence similarity between strains A346^{T}, 3-1745^{T} and members of the genus *Marinobacterium*.

	1*	2*	3*	4*	5*	6*	7*	8*	9*	10*	11*	12*	13*	14	15	16	17	18	19	20	21	22
1*	100	95.9	97.3	96.0	94.6	95.7	93.9	93.3	92.5	92.3	92.2	91.8	92.8	97.3	95.5	96.3	93.5	93.2	93.4	93.3	93.4	92.8
2*	95.9	100	95.7	95.8	93.2	95.5	93.0	92.0	91.6	92.4	92.2	92.4	93.3	97.5	94.8	96.9	93.1	92.1	93.2	92.3	92.4	91.7
3*	97.3	95.7	100	97.5	93.5	97.0	93.4	92.6	92.1	93.2	93.0	93.4	94.2	97.6	96.8	98.1	92.8	92.8	93.4	93.7	93.8	92.8
4*	96.0	95.8	97.5	100	93.6	96.2	93.5	92.1	92.0	93.1	92.9	93.2	94.6	97.7	95.7	98.1	92.7	92.5	93.9	93.8	93.3	92.4
5*	94.6	93.2	93.5	93.6	100	93.2	98.9	93.7	93.9	91.6	91.4	91.1	92.3	94.2	92.0	93.8	94.7	95.4	96.4	91.9	94.2	95.3
6*	95.7	95.5	97.0	96.2	93.2	100	94.0	93.0	92.7	93.9	93.6	94.0	94.5	96.3	97.9	95.8	93.2	93.7	93.8	93.3	94.0	93.4
7*	93.9	93.0	93.4	93.5	98.9	94.0	100	93.5	93.7	91.7	91.4	91.0	92.2	93.4	92.2	93.8	94.4	95.8	97.0	92.1	94.3	95.6
8*	93.3	92.0	92.6	92.1	93.7	93.0	93.5	100	98.4	93.7	93.7	94.3	94.9	92.7	92.7	93.3	93.1	93.9	94.0	91.8	93.2	93.1
9*	92.5	91.6	92.1	92.0	93.9	92.7	93.7	98.4	100	93.7	93.7	98.2	94.4	92.3	92.2	92.8	92.5	93.5	94.2	91.8	92.9	93.2
10*	92.3	92.4	93.2	93.1	91.6	93.9	91.7	93.7	93.7	100	99.6	98.3	98.3	93.2	93.2	93.0	91.7	91.8	92.0	92.6	92.6	91.1
11*	92.2	92.2	93.0	92.9	91.4	93.6	91.4	93.7	93.7	99.6	100	94.3	98.1	93.1	93.1	93.1	91.8	91.7	91.8	92.4	92.4	90.9
12*	91.8	92.4	93.4	93.2	91.1	94.0	91.0	94.3	98.2	98.3	94.3	100	99.0	93.1	92.8	93.7	91.6	91.9	91.9	92.8	92.7	90.9
13*	92.8	93.3	94.2	94.6	92.3	94.5	92.2	94.9	94.4	98.3	98.1	99.0	100	94.2	93.5	94.0	92.5	92.7	93.1	93.2	93.7	92.5
14	97.3	97.5	97.6	97.7	94.2	96.3	93.4	92.7	92.3	93.2	93.1	93.1	94.2	100	95.6	97.8	93.5	92.5	93.7	94.1	93.1	92.4
15	95.5	94.8	96.8	95.7	92.0	97.9	92.2	92.7	92.2	93.2	93.1	92.8	93.5	95.6	100	95.1	92.7	92.1	93.1	92.4	93.1	92.4
16	96.3	96.9	98.1	98.1	93.8	95.8	93.8	93.3	92.8	93.0	93.1	93.7	94.0	97.8	95.1	100	92.6	93.1	94.5	94.0	94.1	93.0
17	93.5	93.1	92.8	92.7	94.7	93.2	94.4	93.1	92.5	91.7	91.8	91.6	92.5	93.5	92.7	92.6	100	93.4	93.9	92.3	93.2	93.2
18	93.2	92.1	92.8	92.5	95.4	93.7	95.8	93.9	93.5	91.8	91.7	91.9	92.7	92.5	92.1	93.1	93.4	100	95.4	92.0	94.3	97.9
19	93.4	93.2	93.4	93.9	96.4	93.8	97.0	94.0	94.2	92.0	91.8	91.9	93.1	93.7	93.1	94.5	93.9	95.4	100	92.6	94.5	95.4
20	93.3	92.3	93.7	93.8	91.9	93.3	92.1	91.8	91.8	92.6	92.4	92.8	93.2	94.1	92.4	94.0	92.3	92.0	92.6	100	91.2	91.5
21	93.4	92.4	93.8	93.3	94.2	94.0	94.3	93.2	92.9	92.6	92.4	92.7	93.7	93.1	93.1	94.1	93.2	94.3	94.5	91.2	100	95.2
22	92.8	91.7	92.8	92.4	95.3	93.4	95.6	93.1	93.2	91.1	90.9	90.9	92.5	92.4	92.4	93.0	93.2	97.9	95.4	91.5	95.2	100

Strains: 1, *M. weihaiensis* A346; 2, *M. marina* 3-1745; 3, *M. maritimum* JCM 15134; 4, *M. sediminicola* CN47; 5, *M. mangrovicola* DSM 27697; 6, *M. halophilum* DSM 17586; 7, *M. lutimaris* DSM 22012; 8, *M. nitratireducens* CGMCC 1.7286; 9, *M. aestuariivivens* DB-1; 10, *M. profundum* PAMC 27536; 11, *M. aestuarii* ST58-10; 12, *M. sedimentorum* KMM 9957; 13, *M. rhizophilum* DSM 18822; 14, *M. stanieri* DSM 7027; 15, *M. georgiense* DSM 11526; 16, *M. coralli* R-40509; 17, *M. marisflavi* IMCC 4074; 18, *M. ramblicola* D7; 19, *M. litorale* DSM 23545; 20, *M. jannaschii* DSM 6295; 21, *M. boryeongense* DMHB-2; 22, *M. zhoushanense* CGMCC 1.15341. *: strain from sediment sample.

Supplementary Table 2. Genome statistics of strains A346^T, 3-1745^T and members of the genus *Marinobacterium*.

	Size (bp)	CheckM Completeness	CheckM Contamination	Genome coverage	GC content (%)	Contig Count	N50	Genes	tRNAs	rRNAs	ncRNAs	GenBank assembly accession
***M. marinum* 3-1745**	**3285871**	**98.71%**	**0.66%**	**150.0x**	**56.4**	**59**	**196986**	**3086**	**68**	**7**	**4**	**GCA_013868415.1**
***M. weihaiensis* A346**	**3539703**	**99.57%**	**0.29%**	**150.0x**	**58.9**	**94**	**177095**	**3351**	**70**	**3**	**4**	**GCA_019061305.1**
M. aestuarii ST58-10	5191608	100.0%	0.07%	154x	58.8	1	5191608	4617	83	18	4	GCA_001651805.1
M. alkalitolerans AK62	3335703	99.57%	0.14%	100.0x	58.2	49	125046	3192	55	5	5	GCA_017776525.1
M.arenosum CAU 1594	4802898	100.0%	2.10%	94.24x	60.8	118	78767	4107	73	5	5	GCA_019795155.1
M. georgiense JCM 21667	3922811	98.71%	0.30%	144x	54.9	52	167945	3742	56	8	5	GCA_900107855.1
M. halophilum DSM 17586	3653180	99.57%	0.44%	278.0x	56.0	49	124972	3443	71	3	4	GCA_003014615.1
M. jannaschii DSM 6295	5174280	100.0%	0.94%	ND	55.2	39	201016	4705	62	8	6	GCA_000620085.1
M. litorale DSM 23545	4378172	98.71%	0.30%	144x	56.4	69	137897	4252	57	9	7	GCA_900107855.1
M. lutimaris DSM 22012	5568156	100.0%	1.38%	150x	57.4	22	869882	5129	69	4	9	GCA_900108065.1
M. mangrovicola DSM 27697	4979440	100.0%	1.14%	191.0x	57.1	17	891038	4518	68	ND	7	GCA_004339595.1
M. nitratireducens CGMCC 1.7286	5546883	99.57%	1.22%	31X	62.1	47	314573	4784	70	3	5	GCA_014645375.1
M. profundum PAMC27536	5637742	99.57%	2.05%	168x	57.2	226	61011	5061	82	5	6	GCA_001528745.1
M. rhizophilum DSM 18822	5360582	100.0%	0.33%	ND	58.5	68	143318	4768	66	6	6	GCA_000378045.1
M. ramblicola D7	4886764	99.93%	1.73%	844.0x	59.2	67	150886	4376	59	1	5	GCA_019084545.1
M. stanieri DSM 7027	4679482	99.57%	0.36%	253x	55.6	26	388460	4380	73	6	5	GCA_900155945.1
M. zhoushanense CGMCC 1.15341	4734355	99.93%	0.93%	31x	58.4	36	383552	4403	73	7	6	GCA_014641945.1
M. sedimentorum KMM 9957	5345811	100%	1.9%	112x	58.5	246	124865	4787	49	4	7	GCA_024267675.1

Supplementary Table 3. The number of core genes, accessory genes, unique genes and exclusively absent genes in the genus *Marinobacterium*.

Genome no.	Organism name	No. of core genes	No. of accessory genes	No. of unique genes	No. of exclusively absent genes
1	***M. marina* 3-1745**	1085	1536	321	15
2	***M. weihaiensis* A346**	1085	1757	318	0
3	*M. aestuarii* ST58-10	1085	2883	353	0
4	*M. alkalitolerans* AK62	1085	1785	210	2
5	*M. arenosum* CAU 1594	1085	1842	1103	12
6	*M. georgiense* DSM 11526	1085	1794	667	6
7	*M. halophilum* DSM 17586	1085	1911	280	1
8	*M. jannaschii* DSM 6295	1085	1712	1741	24
9	*M. litorale* DSM 23545	1085	2119	838	5
10	*M. lutimaris* DSM 22012	1085	3250	545	1
11	*M. mangrovicola* DSM 27697	1085	2967	290	4
12	*M. nitratireducens* CGMCC 1.7286	1085	2495	881	3
13	*M. profundum* PAMC 27536	1085	2927	746	4
14	*M. ramblicola* D7	1085	2639	527	1
15	*M. rhizophilum* DSM 18822	1085	2855	568	1
16	*M. stanieri* DSM 7027	1085	2452	678	1
17	*M. zhoushanense* CGMCC 1.15341	1085	2762	323	3
18	*M. sedimentorum* KMM 9957	1085	3017	462	1

Supplementary Table 4. Description of Module in KEGG databases.

Module	Description
M00001	Glycolysis (Embden-Meyerhof pathway), glucose => pyruvate
M00002	Glycolysis, core module involving three-carbon compounds
M00003	Gluconeogenesis, oxaloacetate => fructose-6P
M00307	Pyruvate oxidation, pyruvate => acetyl-CoA
M00009	Citrate cycle (TCA cycle, Krebs cycle)
M00010	Citrate cycle, first carbon oxidation, oxaloacetate => 2-oxoglutarate
M00011	Citrate cycle, second carbon oxidation, 2-oxoglutarate => oxaloacetate
M00007	Pentose phosphate pathway, non-oxidative phase, fructose 6P => ribose 5P
M00005	PRPP biosynthesis, ribose 5P => PRPP
M00008	Entner-Doudoroff pathway, glucose-6P => glyceraldehyde-3P + pyruvate
M00168	CAM (Crassulacean acid metabolism), dark
M00579	Phosphate acetyltransferase-acetate kinase pathway, acetyl-CoA => acetate
M00175	Nitrogen fixation, nitrogen => ammonia
M00530	Dissimilatory nitrate reduction, nitrate => ammonia
M00529	Denitrification, nitrate => nitrogen
M00176	Assimilatory sulfate reduction, sulfate => H2S
M00595	Thiosulfate oxidation by SOX complex, thiosulfate => sulfate
M00144	NADH:quinone oxidoreductase, prokaryotes
M00149	Succinate dehydrogenase, prokaryotes
M00151	Cytochrome bc1 complex respiratory unit
M00155	Cytochrome c oxidase, prokaryotes
M00153	Cytochrome bd ubiquinol oxidase
M00417	Cytochrome o ubiquinol oxidase
M00156	Cytochrome c oxidase, cbb3-type
M00157	F-type ATPase, prokaryotes and chloroplasts
M00082	Fatty acid biosynthesis, initiation
M00083	Fatty acid biosynthesis, elongation
M00086	beta-Oxidation, acyl-CoA synthesis
M00087	beta-Oxidation
M00088	Ketone body biosynthesis, acetyl-CoA => acetoacetate/3-hydroxybutyrate/acetone
M00093	Phosphatidylethanolamine (PE) biosynthesis, PA => PS => PE
M00048	Inosine monophosphate biosynthesis, PRPP + glutamine => IMP
M00049	Adenine ribonucleotide biosynthesis, IMP => ADP, ATP
M00050	Guanine ribonucleotide biosynthesis, IMP => GDP, GTP
M00052	Pyrimidine ribonucleotide biosynthesis, UMP => UDP/UTP, CDP/CTP
M00053	Pyrimidine deoxyribonuleotide biosynthesis, CDP => dCTP
M00938	Pyrimidine deoxyribonuleotide biosynthesis, UDP => dTTP
M00046	Pyrimidine degradation, uracil => beta-alanine, thymine => 3-aminoisobutanoate
M00020	Serine biosynthesis, glycerate-3P => serine
M00018	Threonine biosynthesis, aspartate => homoserine => threonine
M00555	Betaine biosynthesis, choline => betaine
M00033	Ectoine biosynthesis, aspartate => ectoine

M00919	Ectoine degradation, ectoine => aspartate
M00021	Cysteine biosynthesis, serine => cysteine
M00019	Valine/isoleucine biosynthesis, pyruvate => valine / 2-oxobutanoate => isoleucine
M00570	Isoleucine biosynthesis, threonine => 2-oxobutanoate => isoleucine
M00432	Leucine biosynthesis, 2-oxoisovalerate => 2-oxoisocaproate
M00036	Leucine degradation, leucine => acetoacetate + acetyl-CoA
M00016	Lysine biosynthesis, succinyl-DAP pathway, aspartate => lysine
M00028	Ornithine biosynthesis, glutamate => ornithine
M00844	Arginine biosynthesis, ornithine => arginine
M00015	Proline biosynthesis, glutamate => proline
M00133	Polyamine biosynthesis, arginine => agmatine => putrescine => spermidine
M00026	Histidine biosynthesis, PRPP => histidine
M00045	Histidine degradation, histidine => N-formiminoglutamate => glutamate
M00022	Shikimate pathway, phosphoenolpyruvate + erythrose-4P => chorismate
M00023	Tryptophan biosynthesis, chorismate => tryptophan
M00533	Homoprotocatechuate degradation, homoprotocatechuate => 2-oxohept-3-enedioate
M00027	GABA (gamma-Aminobutyrate) shunt
M00118	Glutathione biosynthesis, glutamate => glutathione
M00063	CMP-KDO biosynthesis
M00364	C10-C20 isoprenoid biosynthesis, bacteria
M00793	dTDP-L-rhamnose biosynthesis
M00548	Benzene degradation, benzene => catechol
M00551	Benzoate degradation, benzoate => catechol / methylbenzoate => methylcatechol
M00637	Anthranilate degradation, anthranilate => catechol
M00568	Catechol ortho-cleavage, catechol => 3-oxoadipate
M00569	Catechol meta-cleavage, catechol => acetyl-CoA / 4-methylcatechol => propanoyl-CoA
M00638	Salicylate degradation, salicylate => gentisate
M00623	Phthalate degradation, phthalate => protocatechuate
M00616	Sulfate-sulfur assimilation

Supplementary Table 5. Description of Carbohydrate-active enzymes family

Classify	Description
AA3_1	cellobiose dehydrogenase (EC 1.1.99.18)
AA3_2	ecdysone oxidase (EC 1.1.3.16); glucose 1-oxidase (EC 1.1.3.4); aryl alcohol oxidase (EC 1.1.3.7); oligosaccharide dehydrogenase (FAD) (EC 1.1.5.-); glucose 1-dehydrogenase (FAD, quinone) (EC 1.1.5.9); pyranose dehydrogenase (EC 1.1.99.29)
AA3_3	alcohol oxidase (EC 1.1.3.13)
AA3_4	pyranose:oxygen 2-oxidoreductase / glucose 2-oxidase (EC 1.1.3.10)
CBM48	Modules of approx. 100 residues with glycogen-binding function, appended to GH13 modules. Also found in the beta subunit (glycogen-binding) of AMP-activated protein kinases (AMPK)
GH108	N-acetylmuramidase (EC 3.2.1.17)
GH18	chitinase (EC 3.2.1.14); lysozyme (EC 3.2.1.17); endo-β-N-acetylglucosaminidase (EC 3.2.1.96); peptidoglycan hydrolase with endo-β-N-acetylglucosaminidase specificity (EC 3.2.1.-); Nod factor hydrolase (EC 3.2.1.-); xylanase inhibitor; concanavalin B; narbonin
GH19	chitinase (EC 3.2.1.14); lysozyme (EC 3.2.1.17); [reducing end] chitinase (EC 3.2.1.-)
GH23	lysozyme type G (EC 3.2.1.17); peptidoglycan lyase (EC 4.2.2.n1) also known in the literature as peptidoglycan lytic transglycosylase; chitinase (EC 3.2.1.14)
GH24	lysozyme (EC 3.2.1.17)
GH25	lysozyme (EC 3.2.1.17)
GH33	sialidase or neuraminidase (EC 3.2.1.18); trans-sialidase (EC 2.4.1.-); anhydrosialidase (EC 4.2.2.15); Kdo hydrolase (EC 3.2.1.-); 2-keto-3-deoxynononic acid hydrolase / KDNase (EC 3.2.1.-)
GH38	α-mannosidase (EC 3.2.1.24); mannosyl-oligosaccharide α-1,2-mannosidase (EC 3.2.1.113); mannosyl-oligosaccharide α-1,3-1,6-mannosidase (EC 3.2.1.114); mannosyl-oligosaccharide α-1,3-mannosidase (EC 3.2.1.-)
GH43_12	xylan 1,4-b-xylosidase (EC 3.2.1.37);a-L-arabinofuranosidase (EC 3.2.1.55)
GH5_12	steryl b-glucosidase (EC 3.2.1.104);b-glucosidase (EC 3.2.1.21);b-glucosylceramidase (EC 3.2.1.45)
GH51	endoglucanase (EC 3.2.1.4); endo-β-1,4-xylanase (EC 3.2.1.8); β-xylosidase (EC 3.2.1.37); α- L-arabinofuranosidase (EC 3.2.1.55); cellobiohydrolase (EC 3.2.1.91)
GH63	processing α-glucosidase (EC 3.2.1.106); α-1,3-glucosidase (EC 3.2.1.84); α-glucosidase (EC 3.2.1.20); mannosylglycerate α-mannosidase / mannosylglycerate hydrolase (EC 3.2.1.170); glucosylglycerate hydrolase (EC 3.2.1.208)
GT2	cellulose synthase (EC 2.4.1.12); chitin synthase (EC 2.4.1.16); dolichyl-phosphate β-D-mannosyltransferase (EC 2.4.1.83); dolichyl-phosphate β-glucosyltransferase (EC 2.4.1.117); N-acetylglucosaminyltransferase (EC 2.4.1.-); N-acetylgalactosaminyltransferase (EC 2.4.1.-); hyaluronan synthase (EC 2.4.1.212); chitin oligosaccharide synthase (EC 2.4.1.-); β-1,3-glucan synthase (EC 2.4.1.34); β-1,4-mannan synthase (EC 2.4.1.-); β-mannosylphosphodecaprenol-mannooligosaccharide α-1,6-mannosyltransferase (EC 2.4.1.199); UDP-Galf: rhamnopyranosyl-N-acetylglucosaminyl-PP-decaprenol β-1,4/1,5-galactofuranosyltransferase (EC 2.4.1.287); UDP-Galf: galactofuranosyl-galactofuranosyl-rhamnosyl-N-acetylglucosaminyl-PP-decaprenol β-1,5/1,6-galactofuranosyltransferase (EC 2.4.1.288); dTDP-L-Rha: N-acetylglucosaminyl-PP-decaprenol α-1,3-L-rhamnosyltransferase (EC 2.4.1.289); alternating β-1,3/4-N-acetylmannan synthase (2.4.1.-); UDP-GlcA: N-acetylglucosaminyl-proteoglycan β-1,4-glucuronosyltransferase (EC 2.4.1.225)
GT25	lipopolysaccharide β-1,4-galactosyltransferase (EC 2.4.1.-); β-1,3-glucosyltransferase (EC 2.4.1.-); β-1,2-glucosyltransferase (EC 2.4.1.-); β-1,2-galactosyltransferase (EC 2.4.1.-); LPS β-1,4-galactosyltransferase (EC 2.4.1.-); occidiofungin β-xylosyltransferase (EC 2.4.2.-); UDP-Gal:procollagen β-galactosyltransferase (EC 2.4.1.50)
GT4	sucrose synthase (EC 2.4.1.13); sucrose-phosphate synthase (EC 2.4.1.14); α-glucosyltransferase (EC 2.4.1.52);

	lipopolysaccharide N-acetylglucosaminyltransferase (EC 2.4.1.56); phosphatidylinositol α-mannosyltransferase (EC 2.4.1.57); GDP-Man: Man1GlcNAc2-PP-dolichol α-1,3-mannosyltransferase (EC 2.4.1.132); GDP-Man: Man3GlcNAc2－PP-dolichol/Man4GlcNAc2-PP-dolichol α-1,2-mannosyltransferase (EC 2.4.1.131); digalactosyldiacylglycerol synthase (EC 2.4.1.141); 1,2-diacylglycerol 3-glucosyltransferase (EC 2.4.1.157); diglucosyl diacylglycerol synthase (EC 2.4.1.208); trehalose phosphorylase (EC 2.4.1.231); NDP-Glc: α-glucose α-glucosyltransferase / α,α-trehalose synthase (EC 2.4.1.245); GDP-Man: Man2GlcNAc2-PP-dolichol α-1,6-mannosyltransferase (EC 2.4.1.257); UDP-GlcNAc: 2-deoxystreptamine α-N-acetylglucosaminyltransferase (EC 2.4.1.283); UDP-GlcNAc: ribostamycin α-N-acetylglucosaminyltransferase (EC 2.4.1.285); UDP-Gal α-galactosyltransferase (EC 2.4.1.-); UDP-Xyl α-xylosyltransferase (EC 2.4.2.-); UDP-GlcA α-glucuronyltransferase (EC 2.4.1.-); UDP-Glc α-glucosyltransferase (EC 2.4.1.-); UDP-GalNAc: GalNAc-PP-Und α-1,3-N-acetylgalactosaminyltransferase (EC 2.4.1.306); UDP-GalNAc: N,N'-diacetylbacillosaminyl-PP-Und α-1,3-N-acetylgalactosaminyltransferase (EC 2.4.1.290); ADP-dependent α-maltose-1-phosphate synthase (2.4.1.342); [retaining] UDP-GlcNAc: polypeptide α-N-acetylglucosaminyltransferase (EC 2.4.1.-); UDP-GlcNAc: α-N-acetylglucosaminyltransferase (EC 2.4.1.-)
GT5	UDP-Glc: glycogen glucosyltransferase (EC 2.4.1.11); ADP-Glc: starch glucosyltransferase (EC 2.4.1.21); NDP-Glc: starch glucosyltransferase (EC 2.4.1.242); UDP-Glc: α-1,3-glucan synthase (EC 2.4.1.183) UDP-Glc: α-1,4-glucan synthase (EC 2.4.1.-)
GT83	undecaprenyl phosphate-α-L-Ara4N: 4-amino-4-deoxy-β-L-arabinosyltransferase (EC 2.4.2.43); dodecaprenyl phosphate-β-galacturonic acid: lipopolysaccharide core α-galacturonosyl transferase (EC 2.4.1.-)
GT94	GDP-Man: GlcA-β-1,2-Man-α-1,3-Glc-β-1,4-Glc-α-1-PP-undecaprenol β-1,4-mannosyltransferase (2.4.1.251)

Supplementary Table 6. Complete list of phenotypic result of API 20E, API ZYM and API 50CHB identification systems (bioMérieux) and Biolog GEN III MicroPlates. 1, A346^T; 2, 3-1745^T; 3, *M. georgiense* JCM 21667^T; 4, *M. stanieri* DSM 7027^T; 5, *M. maritimum* JCM 15134^T.

Strains		1	2	3	4	5
**API 20E**						
ONPG	o-nitrophenyl-β-D-galactopyranoside	−	−	−	−	−
ADH	arginine dihydrolase	−	−	−	−	−
LDC	lysine decarboxylase	−	−	−	−	−
ODC	ornithine decarboxylase	−	−	−	−	−
CIT	citrate utilization	−	−	w	+	+
H2S	H2S production	−	−	−	−	−
URE	Urease	−	−	−	−	−
TDA	tryptophan deaminase	−	−	−	−	−
IND	indole production	−	−	−	−	−
VP	Voges-Proskauer reaction	+	+	+	+	+
GEL	gelatinase	+	+	+	+	+
GLU	glucose	−	−	−	−	−
MAN	mannitol	−	−	−	−	−
INO	inositol	−	−	−	−	−
SOR	sorbitol	−	−	−	−	−
RHA	rhamnol	−	−	−	−	−
SAC	sucrose	−	−	−	−	−
MEL	melibiose	−	−	−	−	−
AMY	amygdalin	−	−	−	−	−
ARA	arabinose	−	−	−	−	−
**API 50CHB**						
GLY	glycerol	−	+	+	+	−
ERY	erythritol	−	−	−	−	−
DARA	D-arabinose	−	−	−	+	−
LARA	L-arabinose	+	−	−	−	−
RIB	D-Ribose	−	+	+	+	+
DXYL	D-xylose	−	−	−	+	−
LXYL	L-xylose	+	−	−	−	−
ADO	D-adonitol	−	−	−	−	−
MDX	Methyl-βD-xylopyranoside	+	−	−	−	+
GAL	D-galactose	−	−	−	+	−
GLU	D-glucose	+	−	−	−	+
FRU	D-fructose	−	−	+	−	−
MNE	D-mannose	−	−	−	−	−
SBE	L-sorbose	−	−	+	−	−
RHA	L-rhamnose	−	+	−	−	−
DUL	dulcitol	−	−	−	−	−

INO	Inositol	−	−	−	−	−
MAN	D-manitol	+	−	−	−	−
SOR	D-sorbitol	−	−	−	−	+
MDM	Methyl-αD-mannopyranoside	−	−	−	+	−
MDG	Methyl-αD-glucopyranoside	−	−	−	−	−
NAG	N-acetyl glucosamine	−	−	−	−	−
AMY	amygdalin	+	−	−	−	−
ARB	arbutin	−	−	−	−	−
ESC	esculin ferric citrate	+	+	+	+	+
SAL	salicin	−	−	−	−	−
CEL	D-cellobiose	+	+	−	−	−
MAL	D-maltose	−	−	−	−	−
LAC	D-lactose (bovine origin)	−	−	−	−	−
MEL	D-melibiose	−	−	−	−	−
SAC	D-saccharose (sucrose)	−	−	−	−	−
TRE	D-trehalose	−	−	−	−	−
INU	inulin	−	−	−	−	−
MLZ	D-melezitose	−	−	−	−	−
RAF	D-raffinose	−	+	−	−	−
AMD	amidon (starch)	−	−	−	−	−
GLYG	glycogen	−	−	−	−	−
XLT	D-xylitol	−	−	−	−	−
GEN	gentiobiose	−	−	−	−	−
TUR	D-turanose	−	−	−	−	−
LYX	D-lyxose	−	−	−	−	−
TAG	D-tagatose	+	+	+	+	+
DFUC	D-fucose	−	−	−	−	−
LFUC	L-fucose	−	−	−	−	−
DARL	D-arbaitol	−	−	−	−	−
LARL	L-arbaitol	−	−	−	−	−
GNT	potassium gluconate	−	−	−	−	−
2KG	potassium 2-ketogluconate	+	+	+	+	+
5KG	potassium 5-ketogluconate	+	+	+	+	+
**Biolog GEN III MicroPlate**						
A1	Negative-control	−	−	−	−	−
A2	Dextrin	−	−	−	w	w
A3	D-Maltose	−	−	−	w	+
A4	D-Trehalose	−	−	−	−	−
A5	D-Cellobiose	−	−	−	−	−
A6	Gentiobiose	−	w	−	−	+
A7	Sucrose	−	−	−	−	−
A8	D-Turanose	−	−	w	w	−
A9	Stachyose	−	−	−	w	−
A10	Positive-control	+	+	+	+	−

A11	PH6	+	+	+	+	+
A12	PH5	w	w	w	+	+
B1	D-Raffinose	−	−	w	w	−
B2	D-Lactose	−	−	−	−	−
B3	D-Melibiose	−	−	−	w	+
B4	β-Methyl-D-Glucoside	−	−	−	−	−
B5	D-Salicin	−	−	−	−	−
B6	N-Acetyl-D-Glucosamine	−	−	−	−	−
B7	N-Acetyl-β-D-Mannosamine	−	−	−	−	−
B8	N-Acetyl-D-Galactosamine	−	−	−	−	−
B9	N-Acetyl Neuraminic Acid	−	−	−	−	−
B10	1% NaCl	+	+	+	+	+
B11	4% NaCl	+	+	+	+	+
B12	8% NaCl	+	+	+	+	+
C1	α-D-Glucose	−	−	w	w	−
C2	D-Mannose	−	−	−	−	−
C3	D-Fructose	−	−	−	w	+
C4	D-Galactose	+	−	−	−	+
C5	3-Methyl Glucose	−	−	−	−	−
C6	D-Fucose	w	+	−	w	+
C7	L-Fucose	w	w	−	w	+
C8	L-Rhamnose	+	w	−	−	+
C9	Inosine	−	−	−	−	−
C10	1% Sodium Lactate	w	+	+	+	w
C11	Fusidic Acid	+	+	+	w	+
C12	D-Serine	+	+	+	w	+
D1	D-Sorbitol	−	−	w	−	−
D2	D-Mannitol	−	−	−	−	−
D3	D-Arabitol	−	−	−	−	−
D4	Myo-Inositol	−	−	−	−	−
D5	Glycerol	−	−	w	−	−
D6	D-Glucose-6-PO4	−	−	−	−	−
D7	D-Fructose-6-PO4	w	w	−	w	−
D8	D-Aspartic Acid	−	−	−	−	−
D9	D-Serine	−	−	+	−	−
D10	Troleandomycin	w	w	w	w	w
D11	Rifamycin SV	+	+	+	+	+
D12	Minocycline	w	+	+	w	+
E1	Gelatin	−	−	−	−	−
E2	Glycyl- L-Proline	−	−	−	+	−
E3	L-Alanine	+	+	+	+	+
E4	L-Arginine	−	−	−	−	+
E5	L-Aspartic Acid	−	−	+	−	−
E6	L-Glutamic Acid		+	+	+	+

E7	L-Histidine	−	−	−	−	−
E8	L-Pyroglutamic Acid	−	+	−	+	−
E9	L-Serine	−	−	−	−	−
E10	Lincomycin	+	+	+	+	+
E11	Guanidine HCl	+	+	+	+	+
E12	Niaproof 4	−	w	+	w	+
F1	Pectin	−	−	−	−	−
F2	D-Galacturonic Acid	−	+	−	+	+
F3	L-Galactonic Acid Lactone	−	w	−	−	+
F4	D-Gluconic Acid	−	−	−	−	−
F5	D-Glucuronic Acid	w	+	w	+	+
F6	Glucuronamide	+	+	+	+	+
F7	Mucic Acid	−	−	−	−	−
F8	Quinic Acid	−	−	−	+	−
F9	D-Saccharic Acid	+	−	−	−	−
F10	Vancomycin	+	+	+	+	+
F11	Tetrazolium-violet	−	+	w	−	w
F12	Tetrazolium Blue	−	+	+	+	+
G1	ρ-Hydroxy-Phenylacectic Acid	+	+	+	+	+
G2	Methyl Pyruvate	+	+	+	+	+
G3	D-Lactic Acid Methyl Ester	−	−	−	−	−
G4	L-Lactic Acid	+	+	+	+	+
G5	Citric Acid	−	−	+	−	+
G6	α-keto-Glutaric Acid	+	+	+	+	+
G7	D-Malic Acid	−	w	w	−	+
G8	L-Malic Acid	+	+	+	+	+
G9	Bromo-Succinic Acid	−	+	+	−	+
G10	Nalidixic Acid	+	+	w	w	+
G11	Lithium Chloride	+	+	+	+	+
G12	Potassium Tellurite	+	+	+	w	+
H1	Tween-40	w	−	−	+	−
H2	γ-Amino-Butyric Acid	−	+	−	+	−
H3	α-Hydroxy-Butyric Acid	+	+	+	+	+
H4	β-Hydroxy- D, L-Butyric Acid	+	+	+	+	+
H5	α-keto-Butyric Acid	+	−	+	+	+
H6	Acetoacetic Acid	w	−	w	+	+
H7	Propionic Acid	+	+	+	+	+
H8	Acetic Acid	+	+	+	+	+
H9	Formic Acid	−	−	−	+	−
H10	Aztreonam	+	+	+	w	+
H11	Sodium Butyrate	+	+	+	+	+
H12	Sodium Bromate	+	+	+	w	+
**API ZYM**						
1	control					

2	alkaline phosphatase	+	+	+	+	+
3	esterase (C4)	+	+	+	+	+
4	esterase lipase (C8)	w	w	w	+	w
5	lipase (C14)	w	w	w	w	w
6	leucine arylamidase	+	+	+	+	+
7	valine arylamidase	w	w	+	+	+
8	cystine arylamidase	w	−	−	−	−
9	trypsin	−	−	−	−	−
10	α-chymotrypsin	−	−	−	−	−
11	acid phosphatase	+	−	+	+	+
12	naphthol-AS-BI-phosphohydrolase	+	+	+	+	+
13	α-galactosidase	−	−	−	−	−
14	β-galactosidase	−	−	−	−	−
15	β-glucuronidase	−	−	−	−	−
16	α-glucosidase	−	−	−	−	−
17	β-glucosidase	w	−	−	−	−
18	N-acetyl-β-glucosaminidase	−	−	w	−	w
19	α-mannosidase	−	−	−	−	−
20	α-fucosidase	−	−	−	−	−
